# Supplementary material for: Survival Outcomes in Older Women with Oestrogen-Receptor-Positive Early-Stage Breast Cancer: Primary Endocrine Therapy vs. Surgery by Comorbidity and Frailty Levels
Source: Cancers (Basel). 2024 Feb 11;16(4):749. doi: 10.3390/cancers16040749 (PMC10886896; doi:10.3390/cancers16040749)
Supplement: Supplementary file 1 [file cancers-16-00749-s001.zip › cancers-2862010-supplementary.pdf]

Supplementary files

Table S1. The Strengthening the Reporting of Observational Studies in Epidemiology (STROBE) Statement checklist 2
Supplementary Section S1. Code development……………………………………….4
Table S2. Medical code of breast cancer in CPRD Gold 5
Table S3. Medical code of breast cancer in CPRD Aurum 7
Table S4. ICD-10 codes of breast cancer in Cancer Registry 9
Table S5. OPCS-4 codes for breast cancer and related operation.................... 10
Table S6. Breast cancer-related medication and BNF codes 11
Table S7. Read terms for Charlson comorbidity index 12
Table S8. ICD-10 codes for identifying comorbidities to calculate the Charlson comorbidity index.. 49
Table S9. ICD-10 codes for frailty score 53
Table S10. Mean value and standardised mean difference of selected covariates in propensity score before and after balance 56
Table S11. Tumour characteristics of the study cohort....................................... 57
Supplementary Section S2.Survival time of study cohort between surgery and PET by levels of frailty 58
Table S12. Competing risk regression of all selected covariates for cohort study60
Figure S2. Cumulative incidence function of competing risk between PET and surgery in three level of hospital frailty risk scores 61
Figure S3. Cumulative incidence function of competing risk in high level of frailty between surgery and PET 62

Table S1. The Strengthening the Reporting of Observational Studies in Epidemiology (STROBE) Statement checklist

	Item No.	Recommendation	Page No
**Title and abstract**	1	(*a*) Indicate the study's design with a commonly used term in the title or the abstract	Title
		(*b*) Provide in the abstract an informative and balanced summary of what was done and what was found	Abstrac
**Introduction**			
Background/rationale	2	Explain the scientific background and rationale for the investigation being reported.	Introduction
Objectives	3	State the specific objectives, including any prespecified hypotheses	Introduction, final paragraph
**Methods**			
Study design	4	Present key elements of study design early in the paper	Method
Setting	5	Describe the setting, locations, and relevant dates, including recruitment periods, exposure, follow-up, and data collection.	Data source
Participants	6	(*a*) Give the eligibility criteria and the sources and methods of selection of participants. Describe methods of follow-up	Study population
		(*b*) For matched studies, give matching criteria and the number of exposed and unexposed	NA
Variables	7	Clearly define all outcomes, exposures, predictors, potential confounders, and effect modifiers. Give diagnostic criteria, if applicable.	Treatment exposure, Outcome measure, covariates
Data sources/ measurement	8*	For each variable of interest, give sources of data and details of assessment methods (measurement). Describe the comparability of assessment methods if there is more than one group.	Covariates
Bias	9	Describe any efforts to address potential sources of bias	Data analysis
Study size	10	Explain how the study size was arrived at	Results, characteristics of the study cohort
Quantitative variables	11	Explain how quantitative variables were handled in the analyses. If applicable, describe which groupings were chosen and why	Covariates
Statistical methods	12	(*a*) Describe all statistical methods, including those used to control for confounding	Data analysis
		(b) Describe any methods used to examine subgroups and interactions	Data analysis
		(c) Explain how missing data were addressed	Data analysis
		(d) If applicable, explain how the loss to follow-up was addressed	
		(e) Describe any sensitivity analyses	Data analysis
**Results**			

Participants	13*	(a) Report numbers of individuals at each stage of the study, e.g., numbers potentially eligible, examined for eligibility, confirmed eligible, included in the study, completing follow-up, and analysed	Results, characteristics of the study cohort
		(b) Give reasons for non-participation at each stage	Figure 2
		(c) Consider the use of a flow diagram	Figure 2
Descriptive data	14*	(a) Give characteristics of study participants (e.g., demographic, clinical, social) and information on exposures and potential confounders	Results, characteristics of the study cohort
		(b) Indicate the number of participants with missing data for each variable of interest	Results, characteristics of the study cohort
		(c) Summarise follow-up time (e.g., average and total amount)	Results, characteristics of the study cohort
Outcome data	15*	Report numbers of outcome events or summary measures over time	Results

Supplementary Section S1. Code development

A population with site-specific cancer was identified from patients with cancer by applying relevant cancer-related codes in CPRD (GOLD and Aurum), HES APC and NCRAS. The cancer-related code list for CPRD was developed by applying cancer-related terms and an algorithm previously developed by the research team to search the CPRD GOLD dictionary (Read codes) and the CPRD Aurum dictionary (SNOMED and EMIS codes) (Figure S1). Two clinicians at the Christie NHS Foundation Trust Manchester (a tertiary cancer hospital in Northwest England) reviewed and confirmed the final code list. The cancer-related ICD-10 code list was applied to NCRAS and HES datasets to identify patients with cancer. Patients were enrolled when the incident cancer (i.e., the first code for cancer) was identified in any linked databases (HES APC and NCRAS).

Figure S1. Procedure for code list development

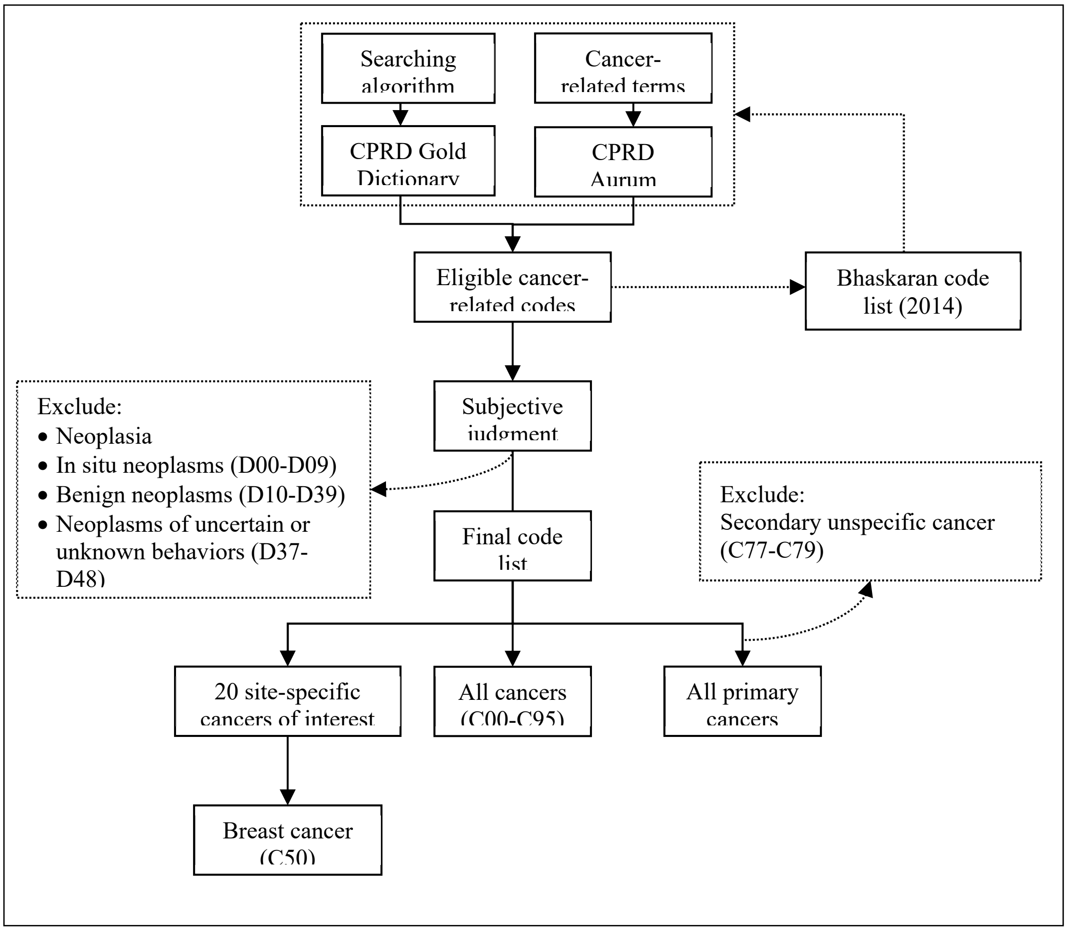

(Note): Bhaskaran code list (2014) [39]

Table S2. Medical code of breast cancer in CPRD Gold

Terms of disease	Medical code
Malignant neoplasm of female breast	3968
Ca female breast	348
Malignant neoplasm of nipple and areola of female breast	26853
Malignant neoplasm of nipple of female breast	23380
Malignant neoplasm of areola of female breast	64686
Malignant neoplasm of nipple or areola of female breast NOS	59831
Malignant neoplasm of central part of female breast	31546
Malignant neoplasm of upper-inner quadrant of female breast	29826
Malignant neoplasm of lower-inner quadrant of female breast	45222
Malignant neoplasm of upper-outer quadrant of female breast	23399
Malignant neoplasm of lower-outer quadrant of female breast	42070
Malignant neoplasm of axillary tail of female breast	20685
Malignant neoplasm, overlapping lesion of breast	49148
Malignant neoplasm of other site of female breast	56715
Malignant neoplasm of ectopic site of female breast	95057
Malignant neoplasm of other site of female breast NOS	38475
Malignant neoplasm of female breast NOS	9470
Malignant neoplasm of male breast	19423
Malignant neoplasm of nipple and areola of male breast	54494
Malignant neoplasm of nipple of male breast	68480
Malignant neoplasm of areola of male breast	67884
Malignant neoplasm of other site of male breast	54202
Malignant neoplasm of ectopic site of male breast	95323
Malignant neoplasm of male breast NOS	48809
Local recurrence of malignant tumour of breast	105488
[M]Infiltrating duct carcinoma	8351
[M]Intraductal papillary adenocarcinoma with invasion	30189
[M]Infiltrating duct and lobular carcinoma	39760
[M]Comedocarcinoma, noninfiltrating	62871
[M]Comedocarcinoma NOS	58131
[M]Juvenile breast carcinoma	40359
[M]Secretory breast carcinoma	67701
[M]Noninfiltrating intraductal papillary adenocarcinoma	102593
[M]Medullary carcinoma with lymphoid stroma	98883
[M]Lobular carcinoma NOS	12427
[M]Infiltrating ductular carcinoma	7319
[M]Inflammatory carcinoma	32472
[M]Paget's disease, mammary	12300
[M]Paget's disease, breast	60803
[M]Paget's disease and infiltrating breast duct carcinoma	42542
[M]Paget's disease and intraductal carcinoma of breast	12480
[M]Cystosarcoma phyllodes NOS	39312

Terms of disease	Medical code
[M]Cystosarcoma phyllodes, malignant	59251
[X]Malignant neoplasm of breast	12499

Table S3. Medical code of breast cancer in CPRD Aurum

Terms of disease	Medical code
Local recurrence of malignant tumour of breast	1803781000006110
[RFC] Breast cancer	907341000006116
Malignant neoplasm of female breast	1210642019
Ca female breast	531851000006119
Ca breast - NOS	990571000006118
Carcinoma breast	880261000006119
Malignant neoplasm of nipple and areola of female breast	289137018
Ca breast - nipple/central	880271000006114
Malignant neoplasm of nipple of female breast	155417012
Malignant neoplasm of areola of female breast	155089014
Malignant neoplasm of nipple or areola of female breast NOS	289140018
Malignant neoplasm of central part of female breast	289141019
Malignant neoplasm of upper-inner quadrant of female breast	289142014
Ca breast-upper,inner quadrant	880281000006112
Malignant neoplasm of lower-inner quadrant of female breast	289143016
Ca breast-lower,inner quadrant	880291000006110
Malignant neoplasm of upper-outer quadrant of female breast	289144010
Ca breast-upper,outer quadrant	880301000006111
Malignant neoplasm of lower-outer quadrant of female breast	289145011
Ca breast-lower,outer quadrant	880311000006114
Malignant neoplasm of axillary tail of female breast	289146012
Ca breast - axillary tail	880321000006118
Malignant neoplasm, overlapping lesion of breast	289147015
Malignant neoplasm of other site of female breast	289148013
Malignant neoplasm of ectopic site of female breast	289149017
Malignant neoplasm of other site of female breast NOS	289150017
Malignant neoplasm of female breast NOS	289151018
Ca breast - NOS	880331000006115
Malignant neoplasm of male breast	155364013
Ca breast - male	880341000006113
Malignant neoplasm of nipple and areola of male breast	289153015
Malignant neoplasm of nipple of male breast	155418019
Malignant neoplasm of areola of male breast	155090017
Malignant neoplasm of nipple or areola of male breast NOS	289156011
Malignant neoplasm of other site of male breast	289157019
Malignant neoplasm of ectopic site of male breast	289158012
Malignant neoplasm of male breast NOS	289159016
Local recurrence of malignant tumour of breast	459378019
[M]Infiltrating duct carcinoma	310311000006110
[M]Intraductal papillary adenocarcinoma with invasion	1232564019
[M]Infiltrating duct and lobular carcinoma	1228259019
[M]Comedocarcinoma, noninfiltrating	307131000006115
[M]Comedocarcinoma NOS	291536017
[M]Juvenile breast carcinoma	310741000006110

Terms of disease	Medical code
[M]Secretory breast carcinoma	316311000006112
[M]Noninfiltrating intraductal papillary adenocarcinoma	314091000006113
[M]Noninfiltrating intracystic carcinoma	314081000006110
[M]Medullary carcinoma with lymphoid stroma	312171000006116
[M]Lobular carcinoma NOS	291540014
[M]Infiltrating ductular carcinoma	310321000006119
[M]Inflammatory carcinoma	310341000006114
[M]Paget's disease, mammary	314711000006117
[M]Paget's disease, breast	314691000006115
[M]Paget's disease and infiltrating breast duct carcinoma	314671000006116
[M]Paget's disease and intraductal carcinoma of breast	1231363012
[M]Cystosarcoma phyllodes NOS	291695012
[M]Cystosarcoma phyllodes, malignant	307441000006115
[X]Malignant neoplasm of breast	292137017

Table S4. ICD-10 codes of breast cancer in Cancer Registry

Disease term	ICD-10 codes
Malignant neoplasm of breast	C50
Malignant neoplasm: Nipple and areola	C50.0
Malignant neoplasm: Central portion of breast	C50.1
Malignant neoplasm: Upper-inner quadrant of breast	C50.2
Malignant neoplasm: Lower-inner quadrant of breast	C50.3
Malignant neoplasm: Upper-outer quadrant of breast	C50.4
Malignant neoplasm: Lower-outer quadrant of breast	C50.5
Malignant neoplasm: Axillary tail of breast	C50.6
Malignant neoplasm: Overlapping lesion of breast	C50.8
Malignant neoplasm: Breast, unspecified	C50.9

Table S5. OPCS-4 codes for breast cancer and related operation

Strategy	OPCS	Term
Surgery	B271	Total mastectomy and excision of both pectoral muscles and part of the chest wall
	B272	Radical mastectomy/total mastectomy and excision of both pectoral muscles NEC.
	B273	Total mastectomy and excision of pectoralis minor muscle
	B274	Total mastectomy NEC, inc toilet and simple mastectomy, extended simple mastectomy.
	B275	Subcutaneous mastectomy
	B276	Skin sparing mastectomy
	B278	Total excision of breast other specified.
	B279	Unspecified, Mastectomy NEC.
	B281	Quadrantectomy of breast
	B282	Partial excision of breast, Partial mastectomy, WLE, includes wedge or segmental excision of breast NEC.
	B283	Excision of lesion of the breast, includes lumpectomy, excision biopsy.
	B284	Re-excision of breast margins
	B285	Wire guided partial excision of breast
	B286	Excision of accessory breast tissue
	B288	Other specified other excision of breast
	B289	Unspecified other excision of breast
	B341	Subareolar excision of mammillary duct
	B342	Excision of mammillary duct NEC
	B343	Excision of lesion of mammillary duct nec. Microdochectomy.
	B352	Excision of nipple
	B353	Extirpation/removal of lesion of nipple.
	B374	Capsulectomy of breast
	B401	Interstitial laser destruction of lesion of breast
	B408	Destruction of lesion of breast, Other specified
	B409	Destruction of lesion of breast, Unspecified

Table S6. Breast cancer-related medication and BNF codes

Type	Code	Description	BNF Code
Drug	Tamoxifen	Anti-oestrogens	0803041S0
	Anastrozole	Aromatase inhibitors	0803041B0
	Letrozole	Aromatase inhibitors	0803041L0
	Exemestane	Aromatase inhibitors	0803041C0

Table S7. Read terms for Charlson comorbidity index

Read/OXMIS term	Disease category	Charlson score weight
HIV disease resulting in unspecified malignant neoplasm	AIDS	6
HIV disease resulting in multiple diseases CE	AIDS	6
[X]Human immunodeficiency virus disease	AIDS	6
HIV disease resulting in mycobacterial infection	AIDS	6
Human immunodeficiency virus with neurological disease	AIDS	6
[X]HIV disease resulting in other non-Hodgkin's lymphoma	AIDS	6
HIV disease resulting/unspcf infectious disease	AIDS	6
Acquired human immunodeficiency virus infection syndrome NOS	AIDS	6
[X]HIV disease resulting in unspecified malignant neoplasm	AIDS	6
[X]HIV disease resulting in multiple infections	AIDS	6
HIV disease resulting in candidiasis	AIDS	6
Human immunodeficiency virus with secondary infection	AIDS	6
[X]HIV disease resulting in other bacterial infections	AIDS	6
[X]Unspecified human immunodeficiency virus [HIV] disease	AIDS	6
[X]HIV disease resulting in other specified conditions	AIDS	6
HIV disease resulting in Burkitt's lymphoma	AIDS	6
[X]HIV disease resulting in other mycoses	AIDS	6
HIV disease resulting in lymphoid interstitial pneumonitis	AIDS	6
Human immunodef virus resulting in other disease	AIDS	6
HIV disease resulting in wasting syndrome	AIDS	6
[X]HIV disease resulting in other viral infections	AIDS	6
Human immunodeficiency virus with constitutional disease	AIDS	6
Acquired immune deficiency syndrome	AIDS	6
[X]HIV disease result/haematological???? abnorms,NEC	AIDS	6
AIDS	AIDS	6
HIV disease resulting in cytomegaloviral disease	AIDS	6
HIV disease resulting in Kaposi's sarcoma	AIDS	6
HIV infection with persistent generalised lymphadenopathy	AIDS	6
HIV disease result/haematological???? abnorms,NEC	AIDS	6
Human immunodeficiency virus with other clinical findings	AIDS	6
Human immunodeficiency virus with secondary cancers	AIDS	6
ACQUIRED IMMUNE DEFICIENCY SYNDROME	AIDS	6
HIV disease resulting in Pneumocystis carinii pneumonia	AIDS	6
[X]HIV disease resulting/unspcf infectious??? disease	AIDS	6
HIV dis reslt/oth mal neopl/lymph,h'matopoetc? tissu	AIDS	6

**Read/OXMIS term**	**Disease category**	**Charlson score weight**
[X]HIV dis reslt/oth mal neopl/lymph,h'matopoetc? tissu	AIDS	6
Asymptomatic human immunodeficiency virus infection	AIDS	6
[X]HIV disease resulting in other malignant neoplasms	AIDS	6
[X]HIV disease resulting/other infectious??? diseases	AIDS	6
HIV disease resulting in multiple infections	AIDS	6
HIV disease resulting in multiple malignant neoplasms	AIDS	6
[X]HIV disease resulting in multiple diseases CE	AIDS	6
Cerebrovascular disease NOS	Cerebrovascular disease	1
Intracranial haemorrhage NOS	Cerebrovascular disease	1
Cerebellar haemorrhage	Cerebrovascular disease	1
Cerebrovascular disease	Cerebrovascular disease	1
Other cerebrovascular disease	Cerebrovascular disease	1
Right sided intracerebral haemorrhage, unspecified	Cerebrovascular disease	1
MENINGEAL HAEMORRHAGE TRAUMATIC	Cerebrovascular disease	1
Precerebral artery occlusion NOS	Cerebrovascular disease	1
Subarachnoid haemorrh from intracranial artery, unspecif	Cerebrovascular disease	1
Subarachnoid haemorrhage from vertebral artery	Cerebrovascular disease	1
Traumatic subdural haemorrhage	Cerebrovascular disease	1
CEREBROVASCULAR DISEASE WITH HYPERTENSIO	Cerebrovascular disease	1
Transient cerebral ischaemia NOS	Cerebrovascular disease	1
Stenosis of precerebral arteries	Cerebrovascular disease	1
Evacuation of intracerebral haematoma NEC	Cerebrovascular disease	1
Other cerebrovascular disease OS	Cerebrovascular disease	1
HAEMORRHAGE INTRACEREBRAL	Cerebrovascular disease	1
[X]Occlusion and stenosis of other cerebral arteries	Cerebrovascular disease	1
Sequelae/other unspecified cerebrovascular diseases	Cerebrovascular disease	1
MENINGEAL HAEMORRHAGE	Cerebrovascular disease	1
Precerebral arterial occlusion	Cerebrovascular disease	1
Transient cerebral ischaemia NOS	Cerebrovascular disease	1
Intracerebral haemorrhage NOS	Cerebrovascular disease	1

Read/OXMIS term	Disease category	Charlson score weight
Generalised ischaemic cerebrovascular disease NOS	Cerebrovascular disease	1
CEREBROVASCULAR DISEASE	Cerebrovascular disease	1
Other transient cerebral ischaemia	Cerebrovascular disease	1
Cerebral degeneration due to cerebrovascular disease	Cerebrovascular disease	1
Occlusion??? of multiple and bilat cerebral arteries	Cerebrovascular disease	1
Subarachnoid haemorrhage	Cerebrovascular disease	1
[X]Cerebrovascular diseases	Cerebrovascular disease	1
HAEMORRHAGE INTRACEREBRAL WITH HYPERTENS	Cerebrovascular disease	1
CVA - cerebrovascular accid due to intracerebral haemorrhage	Cerebrovascular disease	1
Other precerebral artery occlusion	Cerebrovascular disease	1
Subdural haemorrhage NOS	Cerebrovascular disease	1
Open traumatic subarachnoid haemorrhage	Cerebrovascular disease	1
Other specified cerebrovascular disease	Cerebrovascular disease	1
Transient cerebral ischaemia	Cerebrovascular disease	1
[X]Other specified cerebrovascular diseases	Cerebrovascular disease	1
Intracerebral haemorrhage, multiple localized	Cerebrovascular disease	1
[X]Occlusion and stenosis of other precerebral arteries	Cerebrovascular disease	1
H/O: cerebrovascular disease	Cerebrovascular disease	1
[X]Sequelae/other unspecified cerebrovascular diseases	Cerebrovascular disease	1
Subarachnoid haemorrhage from anterior communicating artery	Cerebrovascular disease	1
Subarachnoid haemorrhage from posterior communicating artery	Cerebrovascular disease	1
Sequelae of intracerebral haemorrhage	Cerebrovascular disease	1
[X]Other intracerebral haemorrhage	Cerebrovascular disease	1
Subarachnoid haemorrhage from middle cerebral artery	Cerebrovascular disease	1
Other cerebrovascular disease NOS	Cerebrovascular disease	1
Left sided intracerebral haemorrhage, unspecified	Cerebrovascular disease	1
Intracerebral haemorrhage in hemisphere, unspecified	Cerebrovascular disease	1
Generalised ischaemic cerebrovascular disease NOS	Cerebrovascular disease	1

Read/OXMIS term	Disease category	Charlson score weight
Cerebral infarction due to embolism of cerebral arteries	Cerebrovascular disease	1
[X]Intracerebral haemorrhage in hemisphere, unspecified	Cerebrovascular disease	1
Intracerebral haemorrhage, intraventricular	Cerebrovascular disease	1
Sequelae of subarachnoid haemorrhage	Cerebrovascular disease	1
HAEMORRHAGE INTRACRANIAL	Cerebrovascular disease	1
Traumatic subarachnoid haemorrhage	Cerebrovascular disease	1
Subarachnoid haemorrhage NOS	Cerebrovascular disease	1
HAEMORRHAGE SUBARACHNOID TRAUMATIC	Cerebrovascular disease	1
TRANSIENT CEREBRAL ISCHAEMIA WITH HYPERT	Cerebrovascular disease	1
Subarachnoid haemorrhage following injury	Cerebrovascular disease	1
Multiple and bilateral precerebral arterial occlusion	Cerebrovascular disease	1
Stroke due to intracerebral haemorrhage	Cerebrovascular disease	1
Ruptured berry aneurysm	Cerebrovascular disease	1
Subarachnoid haemorrhage from carotid siphon and bifurcation	Cerebrovascular disease	1
Intracerebral haemorrhage	Cerebrovascular disease	1
Subarachnoid haemorrhage from basilar artery	Cerebrovascular disease	1
Late effects of cerebrovascular disease	Cerebrovascular disease	1
[X]Other subarachnoid haemorrhage	Cerebrovascular disease	1
Closed traumatic subarachnoid haemorrhage	Cerebrovascular disease	1
SUBARACHNOID HAEMORRHAGE WITH HYPERTENSI	Cerebrovascular disease	1
[X]Subarachnoid haemorrhage from other intracranial arteries	Cerebrovascular disease	1
SUBARACHNOID HAEMORRHAGE	Cerebrovascular disease	1
CVA unspecified	Cerebrovascular disease	1
Middle cerebral artery syndrome	Cerebrovascular disease	1
Stroke monitoring	Cerebrovascular disease	1
Cerebral palsy, not congenital or infantile, acute	Cerebrovascular disease	1
Anterior cerebral artery syndrome	Cerebrovascular disease	1
Stroke and cerebrovascular accident unspecified	Cerebrovascular disease	1

Read/OXMIS term	Disease category	Charlson score weight
Left sided CVA	Cerebrovascular disease	1
CVA - Cerebrovascular accident unspecified	Cerebrovascular disease	1
Pure sensory lacunar syndrome	Cerebrovascular disease	1
Brain stem stroke syndrome	Cerebrovascular disease	1
Cerebellar stroke syndrome	Cerebrovascular disease	1
Pure motor lacunar syndrome	Cerebrovascular disease	1
Right sided CVA	Cerebrovascular disease	1
Stroke unspecified	Cerebrovascular disease	1
H/O: CVA/stroke	Cerebrovascular disease	1
Posterior cerebral artery syndrome	Cerebrovascular disease	1
H/O: stroke	Cerebrovascular disease	1
Stroke due to cerebral arterial occlusion	Cerebrovascular disease	1
STROKE	Cerebrovascular disease	1
[X]Hypersensitivity pneumonitis due to other organic dusts	Chronic pulmonary disease	1
Pituitary snuff-takers' disease	Chronic pulmonary disease	1
Flax-dressers' disease	Chronic pulmonary disease	1
Severe asthma attack	Chronic pulmonary disease	1
Allergic alveolitis and pneumonitis NOS	Chronic pulmonary disease	1
Panlobular emphysema	Chronic pulmonary disease	1
BIRD FANCIER'S LUNG	Chronic pulmonary disease	1
ASTHMA ATTACK	Chronic pulmonary disease	1
Sequoiosis (red-cedar asthma)	Chronic pulmonary disease	1
Mild asthma	Chronic pulmonary disease	1
Malt workers' lung	Chronic pulmonary disease	1
Budgerigar-fanciers' lung	Chronic pulmonary disease	1
Other emphysema	Chronic pulmonary disease	1
Talc pneumoconiosis	Chronic pulmonary disease	1
NOCTURNAL ASTHMA	Chronic pulmonary disease	1

Read/OXMIS term	Disease category	Charlson score weight
Asthma	Chronic pulmonary disease	1
Bird-fancier's lung	Chronic pulmonary disease	1
Simple chronic bronchitis	Chronic pulmonary disease	1
Bagassosis	Chronic pulmonary disease	1
Pollen asthma	Chronic pulmonary disease	1
ASTHMA ALLERGIC GRASS	Chronic pulmonary disease	1
EXCISION BRONCHIECTASIS	Chronic pulmonary disease	1
Chest infection - unspecified bronchitis	Chronic pulmonary disease	1
Obstructive chronic bronchitis	Chronic pulmonary disease	1
Severe asthma	Chronic pulmonary disease	1
Post-infective bronchiectasis	Chronic pulmonary disease	1
ASTHMA	Chronic pulmonary disease	1
Asthma causes daytime symptoms most days	Chronic pulmonary disease	1
ASTHMA EPISODIC	Chronic pulmonary disease	1
Other chronic bronchitis NOS	Chronic pulmonary disease	1
Occasional asthma	Chronic pulmonary disease	1
BYSSINOSIS	Chronic pulmonary disease	1
Wood asthma	Chronic pulmonary disease	1
Moderate asthma	Chronic pulmonary disease	1
Bronchiectasis NOS	Chronic pulmonary disease	1
Asthma never causes daytime symptoms	Chronic pulmonary disease	1
EMPHYSEMA PULMONARY	Chronic pulmonary disease	1
Mixed asthma	Chronic pulmonary disease	1
Asthma disturbs sleep weekly	Chronic pulmonary disease	1
FARMERS' LUNG	Chronic pulmonary disease	1
Mushroom workers' lung	Chronic pulmonary disease	1
Simple chronic bronchitis NOS	Chronic pulmonary disease	1
Exercise induced asthma	Chronic pulmonary disease	1

Read/OXMIS term	Disease category	Charlson score weight
ASTHMA ACUTE	Chronic pulmonary disease	1
Asbestosis NOS	Chronic pulmonary disease	1
TRACHEOBRONCHITIS	Chronic pulmonary disease	1
Asthma causing night waking	Chronic pulmonary disease	1
Asthma restricts exercise	Chronic pulmonary disease	1
Silica pneumoconiosis NOS	Chronic pulmonary disease	1
Chronic pulmonary fibrosis due to chemical fumes	Chronic pulmonary disease	1
Asthma - currently dormant	Chronic pulmonary disease	1
Asthma causes daytime symptoms 1 to 2 times per month	Chronic pulmonary disease	1
Hay fever with asthma	Chronic pulmonary disease	1
Extrinsic allergic alveolitis	Chronic pulmonary disease	1
Asthma monitored	Chronic pulmonary disease	1
Status asthmaticus NOS	Chronic pulmonary disease	1
Chronic obstructive pulmonary disease NOS	Chronic pulmonary disease	1
Asthma unspecified	Chronic pulmonary disease	1
Atrophic (senile) emphysema	Chronic pulmonary disease	1
CHRONIC ASTHMA	Chronic pulmonary disease	1
ASTHMA EXACERBATION	Chronic pulmonary disease	1
Chronic bullous emphysema	Chronic pulmonary disease	1
CHRONIC BRONCHITIS WITH EMPHYSEMA	Chronic pulmonary disease	1
Centrilobular emphysema	Chronic pulmonary disease	1
Allergic alveolitis and pneumonitis NOS	Chronic pulmonary disease	1
CHRONIC SPASMODIC BRONCHITIS	Chronic pulmonary disease	1
ASTHMA FREQUENCY REGULARLY	Chronic pulmonary disease	1
ASTHMA POLLEN INITIATED	Chronic pulmonary disease	1
Asthma sometimes restricts exercise	Chronic pulmonary disease	1
Suberosis (cork-handlers' lung)	Chronic pulmonary disease	1
Childhood asthma	Chronic pulmonary disease	1

Read/OXMIS term	Disease category	Charlson score weight
Asthma night-time symptoms	Chronic pulmonary disease	1
Other emphysema NOS	Chronic pulmonary disease	1
Asthma never restricts exercise	Chronic pulmonary disease	1
Silica and silicate pneumoconiosis	Chronic pulmonary disease	1
ASTHMA SEVERITY MILD	Chronic pulmonary disease	1
Bronchitis and pneumonitis due to chemical fumes	Chronic pulmonary disease	1
EXACERBATION OF ASTHMA	Chronic pulmonary disease	1
INTRINSIC ASTHMA	Chronic pulmonary disease	1
ANTHRACOSILICOSIS	Chronic pulmonary disease	1
Obstructive chronic bronchitis NOS	Chronic pulmonary disease	1
MacLeod's unilateral emphysema	Chronic pulmonary disease	1
ASTHMA AND BRONCHITIS	Chronic pulmonary disease	1
ASTHMA FREQUENCY ON EXERCISE ONLY	Chronic pulmonary disease	1
Occupational asthma	Chronic pulmonary disease	1
Other chronic bronchitis	Chronic pulmonary disease	1
Allergic bronchitis NEC	Chronic pulmonary disease	1
PIGEON FANCIER'S LUNG	Chronic pulmonary disease	1
Pneumoconiosis due to inorganic dust NOS	Chronic pulmonary disease	1
Extrinsic asthma with asthma attack	Chronic pulmonary disease	1
Bronchitis unspecified	Chronic pulmonary disease	1
Lung disease due to external agents NOS	Chronic pulmonary disease	1
Asbestosis	Chronic pulmonary disease	1
Attends asthma monitoring	Chronic pulmonary disease	1
Pigeon-fanciers' lung	Chronic pulmonary disease	1
Intrinsic asthma	Chronic pulmonary disease	1
Late onset asthma	Chronic pulmonary disease	1
C/O bronchial catarrh	Chronic pulmonary disease	1
Chronic bronchitis	Chronic pulmonary disease	1

Read/OXMIS term	Disease category	Charlson score weight
BRONCHITIS ACUTE ON CHRONIC	Chronic pulmonary disease	1
Farmers' lung	Chronic pulmonary disease	1
BRONCHITIS PURULENT	Chronic pulmonary disease	1
Brittle asthma	Chronic pulmonary disease	1
Compensatory emphysema	Chronic pulmonary disease	1
Lung disease with diseases EC NOS	Chronic pulmonary disease	1
BRONCHITIS RECURRENT	Chronic pulmonary disease	1
Mucopurulent chronic bronchitis	Chronic pulmonary disease	1
Chronic tracheobronchitis	Chronic pulmonary disease	1
Giant bullous emphysema	Chronic pulmonary disease	1
Asthma disturbing sleep	Chronic pulmonary disease	1
Smokers' cough	Chronic pulmonary disease	1
Chronic pulmonary fibrosis following radiation	Chronic pulmonary disease	1
Asthma confirmed	Chronic pulmonary disease	1
Chronic emphysema due to chemical fumes	Chronic pulmonary disease	1
Intrinsic asthma without status asthmaticus	Chronic pulmonary disease	1
Late-onset asthma	Chronic pulmonary disease	1
Recurrent bronchiectasis	Chronic pulmonary disease	1
Pleural plaque disease due to asbestosis	Chronic pulmonary disease	1
Acute exacerbation of asthma	Chronic pulmonary disease	1
BRONCHITIS ALLERGIC CHRONIC	Chronic pulmonary disease	1
BRONCHIAL ASTHMA	Chronic pulmonary disease	1
Emergency admission, asthma	Chronic pulmonary disease	1
Coal workers' pneumoconiosis	Chronic pulmonary disease	1
ASTHMA HIGH RISK	Chronic pulmonary disease	1
ECZEMA WITH ASTHMA	Chronic pulmonary disease	1
ASTHMA EXERCISE INDUCED	Chronic pulmonary disease	1
H/O: asthma	Chronic pulmonary disease	1

Read/OXMIS term	Disease category	Charlson score weight
Chronic bullous emphysema NOS	Chronic pulmonary disease	1
Chronic catarrhal bronchitis	Chronic pulmonary disease	1
Mixed simple and mucopurulent chronic bronchitis	Chronic pulmonary disease	1
Chronic bronchitis NOS	Chronic pulmonary disease	1
Intrinsic asthma NOS	Chronic pulmonary disease	1
Siderosis	Chronic pulmonary disease	1
Bronchiectasis	Chronic pulmonary disease	1
Emphysematous bronchitis	Chronic pulmonary disease	1
Bird-fancier's lung NOS	Chronic pulmonary disease	1
Intrinsic asthma with status asthmaticus	Chronic pulmonary disease	1
Other allergic alveolitis NOS	Chronic pulmonary disease	1
Stannosis	Chronic pulmonary disease	1
Asthma attack NOS	Chronic pulmonary disease	1
Furriers' lung	Chronic pulmonary disease	1
BRONCHITIS SUBACUTE	Chronic pulmonary disease	1
Asthma attack	Chronic pulmonary disease	1
Asthma causes night symptoms 1 to 2 times per month	Chronic pulmonary disease	1
Emphysema NOS	Chronic pulmonary disease	1
Extrinsic asthma NOS	Chronic pulmonary disease	1
Bronchial asthma	Chronic pulmonary disease	1
Acute vesicular emphysema	Chronic pulmonary disease	1
Asthma daytime symptoms	Chronic pulmonary disease	1
Obliterative bronchiolitis due to chemical fumes	Chronic pulmonary disease	1
[X]Pneumoconiosis due to other dust containing silica	Chronic pulmonary disease	1
STATUS ASTHMATICUS	Chronic pulmonary disease	1
Hay fever with asthma	Chronic pulmonary disease	1
Pneumoconiosis due to other inorganic dust	Chronic pulmonary disease	1
ASTHMA EXERCISE INCLUDED	Chronic pulmonary disease	1

Read/OXMIS term	Disease category	Charlson score weight
ASTHMA SEVERITY MODERATE	Chronic pulmonary disease	1
Massive silicotic fibrosis	Chronic pulmonary disease	1
BRONCHIECTASIS	Chronic pulmonary disease	1
Bronchitis and pneumonitis due to chemical fumes NOS	Chronic pulmonary disease	1
Extrinsic asthma without status asthmaticus	Chronic pulmonary disease	1
CHRONIC BRONCHITIS	Chronic pulmonary disease	1
Hypersensitivity pneumonitis NOS	Chronic pulmonary disease	1
BRONCHITIS OBSTRUCTIVE	Chronic pulmonary disease	1
Asthma causes daytime symptoms 1 to 2 times per week	Chronic pulmonary disease	1
ASTHMA OCCASIONAL	Chronic pulmonary disease	1
Asthma limiting activities	Chronic pulmonary disease	1
Lung disease with diseases EC	Chronic pulmonary disease	1
OBSTRUCTIVE LUNG DISEASE COMPENSATORY	Chronic pulmonary disease	1
Purulent chronic bronchitis	Chronic pulmonary disease	1
Interstitial emphysema	Chronic pulmonary disease	1
OBSTRUCTIVE LUNG DISEASE	Chronic pulmonary disease	1
Pneumoconiosis NOS	Chronic pulmonary disease	1
Mucopurulent chronic bronchitis NOS	Chronic pulmonary disease	1
Subcutaneous emphysema	Chronic pulmonary disease	1
WHEEZING BRONCHIAL	Chronic pulmonary disease	1
[X]Pneumoconiosis due to other specified inorganic dusts	Chronic pulmonary disease	1
Tracheobronchitis NOS	Chronic pulmonary disease	1
Asthma limits walking up hills or stairs	Chronic pulmonary disease	1
Byssinosis	Chronic pulmonary disease	1
Maple bark strippers' lung	Chronic pulmonary disease	1
Bronchitis NOS	Chronic pulmonary disease	1
Asthma treatment compliance unsatisfactory	Chronic pulmonary disease	1
Aspirin induced asthma	Chronic pulmonary disease	1

**Read/OXMIS term**	**Disease category**	**Charlson score weight**
Absent from work or school due to asthma	Chronic pulmonary disease	1
Berylliosis	Chronic pulmonary disease	1
Emphysema	Chronic pulmonary disease	1
Chronic asthmatic bronchitis	Chronic pulmonary disease	1
Asthma prophylactic medication used	Chronic pulmonary disease	1
BRONCHITIS	Chronic pulmonary disease	1
Segmental bullous emphysema	Chronic pulmonary disease	1
Asthma severely restricts exercise	Chronic pulmonary disease	1
Chronic wheezy bronchitis	Chronic pulmonary disease	1
Cannabinosis	Chronic pulmonary disease	1
Fetid chronic bronchitis	Chronic pulmonary disease	1
Allergic asthma NEC	Chronic pulmonary disease	1
LATE ONSET ASTHMA	Chronic pulmonary disease	1
BRONCHITIS ALLERGIC	Chronic pulmonary disease	1
Aluminosis of lung	Chronic pulmonary disease	1
ASBESTOSIS	Chronic pulmonary disease	1
Intrinsic asthma with asthma attack	Chronic pulmonary disease	1
Bauxite fibrosis of lung	Chronic pulmonary disease	1
[X]Other emphysema	Chronic pulmonary disease	1
Detergent asthma	Chronic pulmonary disease	1
Other allergic alveolitis	Chronic pulmonary disease	1
Extrinsic (atopic) asthma	Chronic pulmonary disease	1
Exercise induced asthma	Chronic pulmonary disease	1
CARDIAC ASTHMA	Congestive heart disease	1
Cardiac failure NOS	Congestive heart disease	1
Decompensated cardiac failure	Congestive heart disease	1
Hypertensive heart&renal dis wth (congestive) heart failure	Congestive heart disease	1
HEART FAILURE LEFT-SIDED	Congestive heart disease	1

**Read/OXMIS term**	**Disease category**	**Charlson score weight**
LVF (LEFT VENTRICULAR FAILURE)	Congestive heart disease	1
Weak heart	Congestive heart disease	1
Heart failure annual review	Congestive heart disease	1
MYOCARDIAL FAILURE	Congestive heart disease	1
Congestive cardiomyopathy	Congestive heart disease	1
Compensated cardiac failure	Congestive heart disease	1
Right heart failure	Congestive heart disease	1
Cardiac failure therapy	Congestive heart disease	1
CONGESTIVE CARDIAC FAILURE	Congestive heart disease	1
Congestive cardiac failure	Congestive heart disease	1
Acute heart failure	Congestive heart disease	1
H/O: Heart failure in last year	Congestive heart disease	1
Acute left ventricular failure	Congestive heart disease	1
Heart failure as a complication of care	Congestive heart disease	1
Chronic congestive heart failure	Congestive heart disease	1
Heart failure care plan discussed with patient	Congestive heart disease	1
CONGESTIVE CARDIOMYOPATHY	Congestive heart disease	1
LEFT VENTRICULAR FAILURE ACUTE	Congestive heart disease	1
Left ventricular failure	Congestive heart disease	1
CONGESTIVE HEART FAILURE	Congestive heart disease	1
Heart failure	Congestive heart disease	1
HEART FAILURE ACUTE	Congestive heart disease	1
Congestive heart failure	Congestive heart disease	1
H/O: heart failure	Congestive heart disease	1
WEAK HEART	Congestive heart disease	1
Cardiac failure	Congestive heart disease	1
Heart failure confirmed	Congestive heart disease	1
HYPERTENSION CONGESTIVE HEART FAILURE	Congestive heart disease	1

Read/OXMIS term	Disease category	Charlson score weight
Acute congestive heart failure	Congestive heart disease	1
HEART FAILURE RIGHT-SIDED	Congestive heart disease	1
Admit heart failure emergency	Congestive heart disease	1
CONGESTIVE HEART FAILURE COMPENSATED	Congestive heart disease	1
Heart failure NOS	Congestive heart disease	1
CONGESTIVE HEART FAILURE DECOMPENSATED	Congestive heart disease	1
HEART FAILURE	Congestive heart disease	1
DEMENTIA	Dementia	1
Presenile dementia NOS	Dementia	1
Arteriosclerotic dementia with paranoia	Dementia	1
[X]Alzheimer's dementia unspec	Dementia	1
Uncomplicated presenile dementia	Dementia	1
DEMENTIA ARTERIOSCLEROTIC	Dementia	1
[X]Vascular dementia, unspecified	Dementia	1
[X]Lewy body dementia	Dementia	1
[X]Senile dementia,Alzheimer's type	Dementia	1
Multi infarct dementia	Dementia	1
[X] Senile dementia NOS	Dementia	1
Dementia in conditions EC	Dementia	1
[X]Predominantly cortical dementia	Dementia	1
[X] Senile dementia, depressed or paranoid type	Dementia	1
[X]Dementia in Alzheimer's disease	Dementia	1
SENILE DETERIORATION	Dementia	1
[X] Unspecified dementia	Dementia	1
[X]Presenile dementia,Alzheimer's type	Dementia	1
Arteriosclerotic dementia	Dementia	1
[X]Dementia in other diseases classified elsewhere	Dementia	1
[X]Vascular dementia of acute onset	Dementia	1
[X]Multi-infarct dementia	Dementia	1
DEMENTIA AGGRESSIVE	Dementia	1
Uncomplicated senile dementia	Dementia	1
Arteriosclerotic dementia NOS	Dementia	1
[X]Dementia in other specified diseases classif elsewhere	Dementia	1
Presenile dementia	Dementia	1
[X]Dementia in Alzheimer's disease, unspecified	Dementia	1
[X]Other vascular dementia	Dementia	1
[X]Arteriosclerotic dementia	Dementia	1
[X]Primary degen dementia of Alzheimer's type, senile onset	Dementia	1
Uncomplicated arteriosclerotic dementia	Dementia	1
Senile/presenile dementia	Dementia	1

Read/OXMIS term	Disease category	Charlson score weight
[X]Dementia in Alzheimer's dis, atypical or mixed type	Dementia	1
Senile dementia	Dementia	1
Arteriosclerotic dementia with depression	Dementia	1
Arteriosclerotic dementia with delirium	Dementia	1
SENILE DEMENTIA	Dementia	1
[X]Dementia in Alzheimer's disease with late onset	Dementia	1
[X]Vascular dementia	Dementia	1
PRESENILE DEMENTIA	Dementia	1
[X] Primary degenerative dementia NOS	Dementia	1
H/O: dementia	Dementia	1
[X]Subcortical vascular dementia	Dementia	1
Uncomplicated senile dementia	Dementia	1
[X]Primary degen dementia, Alzheimer's type, presenile onset	Dementia	1
[X]Dementia in Alzheimer's disease with early onset	Dementia	1
[X]Mixed cortical and subcortical vascular dementia	Dementia	1
Unstable diabetes	Diabetes	1
Insulin dependent diabetes mellitus with gangrene	Diabetes	1
Diabetes mellitus, adult onset, with ketoacidosis	Diabetes	1
Lipoatrophic diabetes mellitus	Diabetes	1
Diabetes mellitus, juvenile type, with ketoacidotic coma	Diabetes	1
Non-insulin dependent diabetes mellitus with gangrene	Diabetes	1
Non-insulin-dependent diabetes mellitus without complication	Diabetes	1
Type 2 diabetes mellitus with ulcer	Diabetes	1
Type 2 diabetes mellitus with arthropathy	Diabetes	1
NIDDM - Non-insulin dependent diabetes mellitus	Diabetes	1
Type II diabetes mellitus with arthropathy	Diabetes	1
Diabetes mellitus with unspecified complication	Diabetes	1
Type I diabetes mellitus with ketoacidosis	Diabetes	1
Diabetes mellitus, adult with gangrene	Diabetes	1
GANGRENE DIABETIC	Diabetes	1
Insulin treated Type 2 diabetes mellitus	Diabetes	1
Type I diabetes mellitus maturity onset	Diabetes	1
Diabetes mellitus NOS with hyperosmolar coma	Diabetes	1
HYPOGLYCAEMIC COMA DIABETIC	Diabetes	1
MATURITY ONSET DIABETES MELLITUS INSULIN	Diabetes	1
Diabetic peripheral angiopathy	Diabetes	1
HYPOGLYCAEMIA IN DIABETES MELLITUS	Diabetes	1
Diabetic - poor control	Diabetes	1
Insulin treated Type II diabetes mellitus	Diabetes	1
Type 2 diabetes mellitus with peripheral angiopathy	Diabetes	1
Non-insulin-dependent diabetes mellitus with multiple comps	Diabetes	1
Unstable insulin dependent diabetes mellitus	Diabetes	1

Read/OXMIS term	Disease category	Charlson score weight
Type 2 diabetes mellitus without complication	Diabetes	1
Diabetes mellitus with other specified manifestation	Diabetes	1
Type II diabetes mellitus	Diabetes	1
Type 2 diabetes mellitus with persistent proteinuria	Diabetes	1
UNSTABLE DIABETIC	Diabetes	1
Non-insulin dependent diabetes mellitus with arthropathy	Diabetes	1
Type 2 diabetes mellitus with arthropathy	Diabetes	1
Diabetic on insulin	Diabetes	1
Type II diabetes mellitus with gangrene	Diabetes	1
Pre-existing diabetes mellitus, non-insulin-dependent	Diabetes	1
PRURITUS DIABETIC	Diabetes	1
Type 2 diabetes mellitus with ketoacidosis	Diabetes	1
Unstable type 1 diabetes mellitus	Diabetes	1
Other specified diabetes mellitus with unspecified comps	Diabetes	1
Diabetes mellitus autosomal dominant type 2	Diabetes	1
Diabetes mellitus NOS with peripheral circulatory disorder	Diabetes	1
Type 1 diabetes mellitus with ketoacidotic coma	Diabetes	1
DIABETIC CATARACT	Diabetes	1
Diabetes mellitus, juvenile type, with ketoacidosis	Diabetes	1
Insulin dependent diabetes mellitus	Diabetes	1
DIABETIC ACIDOSIS	Diabetes	1
Unstable type I diabetes mellitus	Diabetes	1
SUGAR DIABETES	Diabetes	1
Diabetes mellitus with ketoacidotic coma	Diabetes	1
Diabetes mellitus with gangrene	Diabetes	1
KETOSIS DIABETIC	Diabetes	1
DIABETES	Diabetes	1
Type 1 diabetes mellitus with ulcer	Diabetes	1
Non-insulin dependent diabetes mellitus with ulcer	Diabetes	1
Other specified diabetes mellitus with other spec comps	Diabetes	1
ABSCESS DIABETIC	Diabetes	1
Type II diabetes mellitus - poor control	Diabetes	1
Malnutrition-related diabetes mellitus with coma	Diabetes	1
Type 2 diabetes mellitus	Diabetes	1
Insulin treated non-insulin dependent diabetes mellitus	Diabetes	1
Diabetic stabilisation	Diabetes	1
Insulin dependent diabetes mellitus	Diabetes	1
Type 1 diabetes mellitus with peripheral angiopathy	Diabetes	1
Type 1 diabetes mellitus with hypoglycaemic coma	Diabetes	1
Type I diabetes mellitus	Diabetes	1
Diabetes mellitus, adult, peripheral circulatory disorder	Diabetes	1
Malnutrition-related diabetes mellitus with ketoacidosis	Diabetes	1

Read/OXMIS term	Disease category	Charlson score weight
Diabetes mellitus, juvenile type, with hyperosmolar coma	Diabetes	1
DIABETIC AMYOTROPHY	Diabetes	1
Type II diabetes mellitus with ulcer	Diabetes	1
Type 2 diabetes mellitus with gangrene	Diabetes	1
KETOACIDOSIS DIABETIC	Diabetes	1
Diabetes mellitus, adult onset, with hyperosmolar coma	Diabetes	1
Type I diabetes mellitus with hypoglycaemic coma	Diabetes	1
Hyperosmolar non-ketotic state in type 2 diabetes mellitus	Diabetes	1
ULCER DIABETIC	Diabetes	1
Diabetic - poor control NOS	Diabetes	1
Insulin treated Type II diabetes mellitus	Diabetes	1
Insulin dependent diabetes mellitus with ulcer	Diabetes	1
Insulin treated Type 2 diabetes mellitus	Diabetes	1
Type 1 diabetes mellitus - poor control	Diabetes	1
Type 1 diabetes mellitus - poor control	Diabetes	1
NIDDM with peripheral circulatory disorder	Diabetes	1
Secondary pancreatic diabetes mellitus	Diabetes	1
Type 1 diabetes mellitus with ketoacidosis	Diabetes	1
Diabetic on insulin and oral treatment	Diabetes	1
Pre-existing diabetes mellitus, insulin-dependent	Diabetes	1
Type 2 diabetes mellitus with ketoacidotic coma	Diabetes	1
Diabetes mellitus with no mention of complication	Diabetes	1
Type 1 diabetes mellitus with gangrene	Diabetes	1
Type 2 diabetes mellitus with hypoglycaemic coma	Diabetes	1
Type 2 diabetes mellitus with peripheral angiopathy	Diabetes	1
Type 1 diabetes mellitus	Diabetes	1
Non-insulin-dependent diabetes mellitus	Diabetes	1
Non-insulin dependent diabetes mellitus with hypoglyca coma	Diabetes	1
Diabetes mellitus NOS with unspecified complication	Diabetes	1
Type 1 diabetes mellitus with hypoglycaemic coma	Diabetes	1
Diabetic annual review	Diabetes	1
Diabetes mellitus, juvenile ??? circulatory disorder	Diabetes	1
Unstable insulin dependent diabetes mellitus	Diabetes	1
Type 1 diabetes mellitus maturity onset	Diabetes	1
Other specified diabetes mellitus with ketoacidosis	Diabetes	1
Type II diabetes mellitus with persistent proteinuria	Diabetes	1
DIABETIC DIARRHOEA	Diabetes	1
HYPEROSMOLAR DIABETIC STATE	Diabetes	1
DIETARY CONTROL DIABETES	Diabetes	1
Type 2 diabetes mellitus - poor control	Diabetes	1
[X]Diabetes mellitus	Diabetes	1
Diabetes mellitus	Diabetes	1
Type 1 diabetes mellitus without complication	Diabetes	1

Read/OXMIS term	Disease category	Charlson score weight
Diabetes mellitus NOS with no mention of complication	Diabetes	1
Diabetes mellitus NOS with ketoacidotic coma	Diabetes	1
Insulin dependent diab mell with peripheral angiopathy	Diabetes	1
Insulin dependent diabetes mellitus with hypoglycaemic coma	Diabetes	1
Diabetes mellitus with ketoacidosis	Diabetes	1
Insulin dependent diabetes mellitus - poor control	Diabetes	1
Type 2 diabetes mellitus - poor control	Diabetes	1
Diabetic - cooperative patient	Diabetes	1
Steroid induced diabetes mellitus without complication	Diabetes	1
Maturity onset diabetes	Diabetes	1
Type 1 diabetes mellitus	Diabetes	1
Admit diabetic emergency	Diabetes	1
LATENT DIABETES	Diabetes	1
H/O: diabetes mellitus	Diabetes	1
Type I diabetes mellitus - poor control	Diabetes	1
Insulin dependent diabetes mellitus	Diabetes	1
Diabetes mellitus, adult onset, no mention of complication	Diabetes	1
Type 2 diabetes mellitus with ulcer	Diabetes	1
Diabetes mellitus, adult onset, unspecified complication	Diabetes	1
Pre-existing diabetes mellitus, unspecified	Diabetes	1
Patient on maximal tolerated therapy for diabetes	Diabetes	1
Diabetes mellitus NOS with other specified manifestation	Diabetes	1
Diabetes mellitus with hyperosmolar coma	Diabetes	1
Malnutrition-related diabetes mellitus	Diabetes	1
IDDM-Insulin dependent diabetes mellitus	Diabetes	1
Diabetes mellitus NOS with ketoacidosis	Diabetes	1
Type 2 diabetes mellitus with hypoglycaemic coma	Diabetes	1
Insulin dependent diabetes mellitus - poor control	Diabetes	1
PRECOMA DIABETIC	Diabetes	1
Type 1 diabetes mellitus with persistent proteinuria	Diabetes	1
DIABETES MELLITUS INSULIN DEPENDANT	Diabetes	1
Diabetes mellitus induced by non-steroid drugs	Diabetes	1
Diabetes mellitus with peripheral circulatory disorder	Diabetes	1
Maturity onset diabetes in youth type 2	Diabetes	1
IDDM with peripheral circulatory disorder	Diabetes	1
Type II diabetes mellitus with peripheral angiopathy	Diabetes	1
Type II diabetes mellitus with hypoglycaemic coma	Diabetes	1
Type 1 diabetes mellitus with persistent microalbuminuria	Diabetes	1
Non-insulin dependent diabetes mellitus	Diabetes	1
Type 2 diabetes mellitus with persistent microalbuminuria	Diabetes	1
[X]Other specified diabetes mellitus	Diabetes	1

Read/OXMIS term	Disease category	Charlson score weight
Type 2 diabetes mellitus	Diabetes	1
Other specified diabetes mellitus with coma	Diabetes	1
Type II diabetes mellitus	Diabetes	1
Type I diabetes mellitus with ulcer	Diabetes	1
Type I diabetes mellitus with ketoacidotic coma	Diabetes	1
Non-insulin dependant diabetes mellitus - poor control	Diabetes	1
COMA DIABETIC	Diabetes	1
Diabetes mellitus, adult, other specified manifestation	Diabetes	1
Type I diabetes mellitus	Diabetes	1
Secondary diabetes mellitus	Diabetes	1
Type II diabetes mellitus - poor control	Diabetes	1
Diabetic - good control	Diabetes	1
Diabetes mellitus, juvenile type, no mention of complication	Diabetes	1
Diabetic cataract	Diabetes with complications	2
Type 2 diabetes mellitus with neurological complications	Diabetes with complications	2
Diabetes mellitus, juvenile type, with renal manifestation	Diabetes with complications	2
Type II diabetes mellitus with ophthalmic complications	Diabetes with complications	2
O/E - right eye background diabetic retinopathy	Diabetes with complications	2
Type I diabetes mellitus with polyneuropathy	Diabetes with complications	2
Insulin dependent diabetes mellitus with arthropathy	Diabetes with complications	2
Non-insulin dependent diabetes mellitus with nephropathy	Diabetes with complications	2
Insulin dependent diabetes mellitus with mononeuropathy	Diabetes with complications	2
Diabetes mellitus, adult onset, neurological manifestation	Diabetes with complications	2
Insulin-dependent diabetes mellitus with renal complications	Diabetes with complications	2
Non-insulin dependent d m with neuropathic arthropathy	Diabetes with complications	2
Insulin-dependent diabetes mellitus with neurological comps	Diabetes with complications	2
Type 2 diabetes mellitus with ophthalmic complications	Diabetes with complications	2
O/E - left eye background diabetic retinopathy	Diabetes with complications	2
Type II diabetes mellitus with renal complications	Diabetes with complications	2
Preproliferative diabetic retinopathy	Diabetes with complications	2
Diabetes mellitus, juvenile type, ophthalmic manifestation	Diabetes with complications	2
Type 2 diabetes mellitus with gastroparesis	Diabetes with complications	2
Non-insulin-dependent diabetes mellitus with ophthalm comps	Diabetes with complications	2

**Read/OXMIS term**	**Disease category**	**Charlson score weight**
NEUROPATHY DIABETIC	Diabetes with complications	2
Type 2 diabetes mellitus with diabetic cataract	Diabetes with complications	2
Type 1 diabetes mellitus with retinopathy	Diabetes with complications	2
Diabetes mellitus with polyneuropathy	Diabetes with complications	2
Diabetic retinopathy	Diabetes with complications	2
Type 2 diabetes mellitus with retinopathy	Diabetes with complications	2
Non-insulin dependent diabetes mellitus with polyneuropathy	Diabetes with complications	2
Non proliferative diabetic retinopathy	Diabetes with complications	2
Other specified diabetes mellitus with ophthalmic complicatn	Diabetes with complications	2
Type II diabetes mellitus with diabetic cataract	Diabetes with complications	2
Type 2 diabetes mellitus with polyneuropathy	Diabetes with complications	2
Type 2 diabetes mellitus with mononeuropathy	Diabetes with complications	2
Type II diabetes mellitus with retinopathy	Diabetes with complications	2
Type 1 diabetes mellitus with neurological complications	Diabetes with complications	2
Type I diabetes mellitus with mononeuropathy	Diabetes with complications	2
Non-insulin-dependent diabetes mellitus with retinopathy	Diabetes with complications	2
Myasthenic syndrome due to diabetic amyotrophy	Diabetes with complications	2
Diabetes mellitus NOS with neurological manifestation	Diabetes with complications	2
Other specified diabetes mellitus with renal complications	Diabetes with complications	2
O/E - left eye proliferative diabetic retinopathy	Diabetes with complications	2
Type I diabetes mellitus with retinopathy	Diabetes with complications	2
Diabetic maculopathy	Diabetes with complications	2
DIABETIC GLOMERULOSCLEROSIS	Diabetes with complications	2
Type 2 diabetes mellitus with nephropathy	Diabetes with complications	2
Diabetic amyotrophy	Diabetes with complications	2
Kimmelstiel - Wilson disease	Diabetes with complications	2
Insulin dependent diabetes mellitus with nephropathy	Diabetes with complications	2
Type 1 diabetes mellitus with neurological complications	Diabetes with complications	2

**Read/OXMIS term**	**Disease category**	**Charlson score weight**
Insulin-dependent diabetes mellitus with ophthalmic comps	Diabetes with complications	2
Diabetic mononeuropathy	Diabetes with complications	2
Diabetic neuropathy	Diabetes with complications	2
Advanced diabetic maculopathy	Diabetes with complications	2
Type II diabetes mellitus with renal complications	Diabetes with complications	2
O/E - left eye stable treated prolif diabetic retinopathy	Diabetes with complications	2
Type 2 diabetes mellitus with nephropathy	Diabetes with complications	2
Type 1 diabetes mellitus with diabetic cataract	Diabetes with complications	2
Type 2 diabetes mellitus with diabetic cataract	Diabetes with complications	2
Type I diabetes mellitus with nephropathy	Diabetes with complications	2
Type II diabetes mellitus with polyneuropathy	Diabetes with complications	2
Type 2 diabetes mellitus with neurological complications	Diabetes with complications	2
Type 1 diabetes mellitus with mononeuropathy	Diabetes with complications	2
Type 2 diabetes mellitus with neuropathic arthropathy	Diabetes with complications	2
High risk non proliferative diabetic retinopathy	Diabetes with complications	2
Insulin dependent diabetes mellitus with retinopathy	Diabetes with complications	2
Type 1 diabetes mellitus with polyneuropathy	Diabetes with complications	2
CHARCOT'S DIABETIC ARTHROPATHY	Diabetes with complications	2
Polyneuropathy in disease NOS	Diabetes with complications	2
Type 2 diabetes mellitus with ophthalmic complications	Diabetes with complications	2
DIABETIC NEPHROPATHY	Diabetes with complications	2
O/E - diabetic maculopathy present both eyes	Diabetes with complications	2
Type 2 diabetes mellitus with renal complications	Diabetes with complications	2
Non-insulin-dependent diabetes mellitus with renal comps	Diabetes with complications	2
Non-insulin-dependent diabetes mellitus with neuro comps	Diabetes with complications	2
High risk proliferative diabetic retinopathy	Diabetes with complications	2
Diabetes mellitus with neuropathy	Diabetes with complications	2
O/E - left eye preproliferative diabetic retinopathy	Diabetes with complications	2

**Read/OXMIS term**	**Disease category**	**Charlson score weight**
Non-insulin depend diabetes mellitus with diabetic cataract	Diabetes with complications	2
Type 2 diabetes mellitus with retinopathy	Diabetes with complications	2
Insulin dependent diabetes mellitus with polyneuropathy	Diabetes with complications	2
Type I diabetes mellitus with neurological complications	Diabetes with complications	2
Type 1 diabetes mellitus with renal complications	Diabetes with complications	2
Type II diabetes mellitus with neuropathic arthropathy	Diabetes with complications	2
Diabetes mellitus with neurological manifestation	Diabetes with complications	2
Type II diabetes mellitus with retinopathy	Diabetes with complications	2
Diabetic polyneuropathy	Diabetes with complications	2
O/E - right eye stable treated prolif diabetic retinopathy	Diabetes with complications	2
Diabetic retinopathy NOS	Diabetes with complications	2
Diabetes mellitus NOS with ophthalmic manifestation	Diabetes with complications	2
Diabetes mellitus, adult onset, ophthalmic manifestation	Diabetes with complications	2
Type 1 diabetes mellitus with nephropathy	Diabetes with complications	2
Type I diabetes mellitus with diabetic cataract	Diabetes with complications	2
Type I diabetes mellitus with renal complications	Diabetes with complications	2
Type II diabetes mellitus with nephropathy	Diabetes with complications	2
Type 1 diabetes mellitus with renal complications	Diabetes with complications	2
Type II diabetes mellitus with mononeuropathy	Diabetes with complications	2
Diabetic amyotrophy	Diabetes with complications	2
Type II diabetes mellitus with nephropathy	Diabetes with complications	2
Type 1 diabetes mellitus with gastroparesis	Diabetes with complications	2
Non-insulin dependent diabetes mellitus with mononeuropathy	Diabetes with complications	2
Insulin dependent diabetes mellitus with diabetic cataract	Diabetes with complications	2
Diabetic nephropathy	Diabetes with complications	2
O/E - right eye preproliferative diabetic retinopathy	Diabetes with complications	2
Type 1 diabetes mellitus with neuropathic arthropathy	Diabetes with complications	2
Type II diabetes mellitus with polyneuropathy	Diabetes with complications	2

Read/OXMIS term	Disease category	Charlson score weight
Other specified diabetes mellitus with neurological comps	Diabetes with complications	2
KIMMELSTIEL- WILSON DISEASE/SYNDROME	Diabetes with complications	2
Type 2 diabetes mellitus with neuropathic arthropathy	Diabetes with complications	2
Type 2 diabetes mellitus with renal complications	Diabetes with complications	2
Diabetes mellitus with ophthalmic manifestation	Diabetes with complications	2
Proliferative diabetic retinopathy	Diabetes with complications	2
Type II diabetes mellitus with neurological complications	Diabetes with complications	2
Diabetes mellitis with nephropathy NOS	Diabetes with complications	2
Type 1 diabetes mellitus with ophthalmic complications	Diabetes with complications	2
Flaccid paraplegia	Hemiplegia	2
SPASTIC PARAPLEGIA	Hemiplegia	2
Left hemiplegia	Hemiplegia	2
O/E - paraplegia	Hemiplegia	2
HYPERTENSIVE HEMIPLEGIA	Hemiplegia	2
PARAPLEGIA	Hemiplegia	2
Hemiplegia NOS	Hemiplegia	2
Congenital paraplegia	Hemiplegia	2
O/E - hemiplegia	Hemiplegia	2
HEMIPLEGIA FLACCID	Hemiplegia	2
HEMIPLEGIA LEFT	Hemiplegia	2
Spastic hemiplegia	Hemiplegia	2
Right hemiplegia	Hemiplegia	2
Paraplegia - congenital	Hemiplegia	2
Spastic paraplegia	Hemiplegia	2
HEMIPLEGIA WITH HYPERTENSION	Hemiplegia	2
Paraplegia	Hemiplegia	2
PARALYSIS HEMIPLEGIA	Hemiplegia	2
Hereditary spastic paraplegia	Hemiplegia	2
HEMIPLEGIA RIGHT	Hemiplegia	2
Flaccid hemiplegia	Hemiplegia	2
Hemiplegia	Hemiplegia	2
SPASTIC HEMIPLEGIA	Hemiplegia	2
Secondary biliary cirrhosis	Mild liver disease	1
Fatty portal cirrhosis	Mild liver disease	1
Biliary cirrhosis NOS	Mild liver disease	1
Chronic hepatitis	Mild liver disease	1
Chronic hepatitis unspecified	Mild liver disease	1
Multilobular portal cirrhosis	Mild liver disease	1
Toxic liver disease with fibrosis and cirrhosis of liver	Mild liver disease	1
Pigmentary cirrhosis of liver	Mild liver disease	1

Read/OXMIS term	Disease category	Charlson score weight
Laennec's cirrhosis, non-alcoholic	Mild liver disease	1
LIVER CIRRHOSIS	Mild liver disease	1
Chronic alcoholic hepatitis	Mild liver disease	1
Macronodular cirrhosis of liver	Mild liver disease	1
Chronic active hepatitis	Mild liver disease	1
Unilobular portal cirrhosis	Mild liver disease	1
HEPATITIS CHRONIC	Mild liver disease	1
SECONDARY BILIARY CIRRHOSIS (LIVER)	Mild liver disease	1
Portal cirrhosis unspecified	Mild liver disease	1
Non-alcoholic cirrhosis NOS	Mild liver disease	1
Alcoholic cirrhosis of liver	Mild liver disease	1
Pipe-stem portal cirrhosis	Mild liver disease	1
Portal cirrhosis	Mild liver disease	1
Diffuse nodular cirrhosis	Mild liver disease	1
PRIMARY BILIARY CIRRHOSIS (LIVER)	Mild liver disease	1
HEPATITIS CHRONIC ACTIVE	Mild liver disease	1
CIRRHOSIS	Mild liver disease	1
MICRONODULAR CIRRHOSIS	Mild liver disease	1
Xanthomatous portal cirrhosis	Mild liver disease	1
Cirrhosis of liver NOS	Mild liver disease	1
Recurrent hepatitis	Mild liver disease	1
[X]Other and unspecified cirrhosis of liver	Mild liver disease	1
Cirrhosis and chronic liver disease	Mild liver disease	1
Syphilitic portal cirrhosis	Mild liver disease	1
Glycogenosis with hepatic cirrhosis	Mild liver disease	1
CIRRHOSIS ALCOHOLIC	Mild liver disease	1
Cryptogenic cirrhosis of liver	Mild liver disease	1
Primary biliary cirrhosis	Mild liver disease	1
Biliary cirrhosis	Mild liver disease	1
Cirrhosis - non alcoholic	Mild liver disease	1
Hepatitis unspecified	Mild liver disease	1
Cardiac portal cirrhosis	Mild liver disease	1
Chronic persistent hepatitis	Mild liver disease	1
Acute yellow atrophy	Mild liver disease	1
Toxic portal cirrhosis	Mild liver disease	1
MACRONODULAR CIRRHOSIS	Mild liver disease	1
HEPATITIS CHRONIC AGGRESSIVE	Mild liver disease	1
Portal fibrosis without cirrhosis	Mild liver disease	1
Chronic aggressive hepatitis	Mild liver disease	1
Subacute yellow atrophy	Mild liver disease	1
Postnecrotic cirrhosis of liver	Mild liver disease	1
Florid cirrhosis	Mild liver disease	1
Chronic hepatitis NOS	Mild liver disease	1
Oesophageal varices in alcoholic cirrhosis of the liver	Mod liver disease	3
Liver abscess and chronic liver disease causing sequelae NOS	Mod liver disease	3

Read/OXMIS term	Disease category	Charlson score weight
Oesophageal varices without bleeding	Mod liver disease	3
[X]Oesophageal varices in diseases classified elsewhere	Mod liver disease	3
Other sequelae of chronic liver disease	Mod liver disease	3
Oesophageal varices with bleeding in diseases EC	Mod liver disease	3
SYNDROME HEPATORENAL	Mod liver disease	3
Hepatorenal syndrome	Mod liver disease	3
PORTAL HYPERTENSION	Mod liver disease	3
Oesophageal varices with bleeding	Mod liver disease	3
Oesophageal varices in diseases EC	Mod liver disease	3
Hepatic coma	Mod liver disease	3
Oesophageal varices NOS	Mod liver disease	3
Oesophageal varices	Mod liver disease	3
Portal hypertension	Mod liver disease	3
Oesophageal varices in cirrhosis of the liver	Mod liver disease	3
Other specified viral hepatitis with hepatic coma NOS	Mod liver disease	3
Rigid oesophagoscopic injection sclerotherapy oesoph varices	Mod liver disease	3
HEPATIC COMA	Mod liver disease	3
Oesophageal varices without bleeding in diseases EC	Mod liver disease	3
Oesophageal varices in diseases EC NOS	Mod liver disease	3
Other specified anterior myocardial infarction	Myocardial infarction	1
Acute myocardial infarction NOS	Myocardial infarction	1
Heart attack	Myocardial infarction	1
MI - acute myocardial infarction	Myocardial infarction	1
Lateral myocardial infarction NOS	Myocardial infarction	1
THROMBOSIS CORONARY	Myocardial infarction	1
Acute subendocardial infarction	Myocardial infarction	1
H/O: Myocardial infarction in last year	Myocardial infarction	1
Other acute myocardial infarction	Myocardial infarction	1
Acute inferoposterior infarction	Myocardial infarction	1
CORONARY INFARCTION	Myocardial infarction	1
Acute myocardial infarction	Myocardial infarction	1
Acute anterolateral infarction	Myocardial infarction	1
Personal history of myocardial infarction	Myocardial infarction	1
Acute ST segment elevation myocardial infarction	Myocardial infarction	1
Cardiac rupture following myocardial infarction (MI)	Myocardial infarction	1
Acute non-ST segment elevation myocardial infarction	Myocardial infarction	1
Acute non-Q wave infarction	Myocardial infarction	1
Acute anteroseptal infarction	Myocardial infarction	1
Old myocardial infarction	Myocardial infarction	1
Silent myocardial infarction	Myocardial infarction	1
Healed myocardial infarction	Myocardial infarction	1
Coronary thrombosis	Myocardial infarction	1
HEART ATTACK	Myocardial infarction	1
Other acute myocardial infarction NOS	Myocardial infarction	1

**Read/OXMIS term**	**Disease category**	**Charlson score weight**
Acute inferolateral infarction	Myocardial infarction	1
Acute duodenal ulcer unspecified	Peptic ulcer disease	1
Endoscopic injection haemostasis of gastric ulcer	Peptic ulcer disease	1
Unspecified gastrojejunal ulcer with obstruction	Peptic ulcer disease	1
Peptic ulcer - (PU) site unspecified	Peptic ulcer disease	1
ULCER STOMACH PERFORATED	Peptic ulcer disease	1
Peptic ulcer of oesophagus	Peptic ulcer disease	1
ULCER GASTROJEJUNAL	Peptic ulcer disease	1
Unspecified duodenal ulcer NOS	Peptic ulcer disease	1
EXCISION PEPTIC ULCER	Peptic ulcer disease	1
Pyloric ulcer	Peptic ulcer disease	1
ULCER GASTRIC PERFORATED	Peptic ulcer disease	1
Chronic gastrojejunal ulcer with perforation	Peptic ulcer disease	1
Acute gastric ulcer	Peptic ulcer disease	1
Unspecified duodenal ulcer	Peptic ulcer disease	1
Chronic duodenal ulcer unspecified	Peptic ulcer disease	1
Bleeding acute gastric ulcer	Peptic ulcer disease	1
Unspec gastric ulcer; unspec haemorrhage and/or perforation	Peptic ulcer disease	1
ULCER DUODENUM	Peptic ulcer disease	1
Chronic gastric ulcer unspecified	Peptic ulcer disease	1
Chronic gastric ulcer with haemorrhage	Peptic ulcer disease	1
Stomal ulcer	Peptic ulcer disease	1
Chronic peptic ulcer unspecified	Peptic ulcer disease	1
Acute gastric ulcer unspecified	Peptic ulcer disease	1
Peptic ulcer NOS	Peptic ulcer disease	1
Prepyloric ulcer	Peptic ulcer disease	1
Acute gastric ulcer with haemorrhage and perforation	Peptic ulcer disease	1
Operation on gastric ulcer NOS	Peptic ulcer disease	1
Unspecified gastrojejunal ulcer with haemorrhage	Peptic ulcer disease	1
Chronic duodenal ulcer with obstruction	Peptic ulcer disease	1
Acute peptic ulcer NOS	Peptic ulcer disease	1
Unspecified duodenal ulcer with obstruction	Peptic ulcer disease	1
Unspecified gastric ulcer with haemorrhage	Peptic ulcer disease	1
Acute gastrojejunal ulcer with obstruction	Peptic ulcer disease	1
Unspecified duodenal ulcer without mention of complication	Peptic ulcer disease	1
Duodenal ulcer disease	Peptic ulcer disease	1
Chronic duodenal ulcer without mention of complication	Peptic ulcer disease	1
Unspecified gastrojejunal ulcer with perforation	Peptic ulcer disease	1
Gastric ulcer - (GU)	Peptic ulcer disease	1
Chronic gastrojejunal ulcer unspecified	Peptic ulcer disease	1
ULCER GASTROJEJUNAL PERFORATED	Peptic ulcer disease	1
Chronic gastrojejunal ulcer with haemorrhage and perforation	Peptic ulcer disease	1
Unspecified gastric ulcer with obstruction	Peptic ulcer disease	1

Read/OXMIS term	Disease category	Charlson score weight
Unspecified peptic ulcer	Peptic ulcer disease	1
Chronic peptic ulcer	Peptic ulcer disease	1
Acute duodenal ulcer NOS	Peptic ulcer disease	1
Acute gastrojejunal ulcer without mention of complication	Peptic ulcer disease	1
Acute gastric ulcer with perforation	Peptic ulcer disease	1
Chronic gastric ulcer with obstruction	Peptic ulcer disease	1
Chronic duodenal ulcer	Peptic ulcer disease	1
Unspec gastrojejunal ulcer; unspec haemorrhage/perforation	Peptic ulcer disease	1
Gastrocolic ulcer	Peptic ulcer disease	1
Acute peptic ulcer	Peptic ulcer disease	1
Unspecified gastric ulcer with perforation	Peptic ulcer disease	1
Acute duodenal ulcer with haemorrhage and perforation	Peptic ulcer disease	1
ULCER PEPTIC DUODENUM	Peptic ulcer disease	1
Operations on duodenal ulcer	Peptic ulcer disease	1
Acute duodenal ulcer with haemorrhage	Peptic ulcer disease	1
ULCER PEPTIC DUODENUM PERFORATED	Peptic ulcer disease	1
Unspecified peptic ulcer with haemorrhage and perforation	Peptic ulcer disease	1
Unspec gastrojejunal ulcer with haemorrhage and perforation	Peptic ulcer disease	1
ULCER PEPTIC STOMACH PERFORATED	Peptic ulcer disease	1
Chronic peptic ulcer with haemorrhage and perforation	Peptic ulcer disease	1
Bleeding chronic gastric ulcer	Peptic ulcer disease	1
Closure of perforated gastric ulcer	Peptic ulcer disease	1
Acute duodenal ulcer with perforation	Peptic ulcer disease	1
Acute peptic ulcer with perforation	Peptic ulcer disease	1
Suture of ulcer of stomach NEC	Peptic ulcer disease	1
Acute gastrojejunal ulcer with haemorrhage	Peptic ulcer disease	1
Unspecified duodenal ulcer with perforation	Peptic ulcer disease	1
Chronic gastric ulcer NOS	Peptic ulcer disease	1
ULCER DUODENAL RECURRANCE	Peptic ulcer disease	1
Unspecified gastric ulcer NOS	Peptic ulcer disease	1
Gastrojejunal ulcer NOS	Peptic ulcer disease	1
Unspecified gastrojejunal ulcer without mention complication	Peptic ulcer disease	1
Gastric ulcer NOS	Peptic ulcer disease	1
Unspecified gastrojejunal ulcer	Peptic ulcer disease	1
Chronic gastrojejunal ulcer NOS	Peptic ulcer disease	1
Unspec peptic ulcer; unspec haemorrhage and/or perforation	Peptic ulcer disease	1
Balfour excision of gastric ulcer	Peptic ulcer disease	1
Unspec duodenal ulcer; unspec haemorrhage and/or perforation	Peptic ulcer disease	1
Chronic gastric ulcer without mention of complication	Peptic ulcer disease	1
Acute gastric ulcer without mention of complication	Peptic ulcer disease	1
Acute peptic ulcer without mention of complication	Peptic ulcer disease	1

Read/OXMIS term	Disease category	Charlson score weight
ULCER PEPTIC PERFORATED	Peptic ulcer disease	1
Chronic gastric ulcer with haemorrhage and perforation	Peptic ulcer disease	1
Acute gastrojejunal ulcer with haemorrhage and perforation	Peptic ulcer disease	1
Unspecified peptic ulcer without mention of complication	Peptic ulcer disease	1
Gastrojejunal ulcer (GJU)	Peptic ulcer disease	1
Unspecified gastric ulcer	Peptic ulcer disease	1
Peptic ulcer symptoms	Peptic ulcer disease	1
Acute duodenal ulcer	Peptic ulcer disease	1
Acute peptic ulcer with obstruction	Peptic ulcer disease	1
Chronic gastrojejunal ulcer with obstruction	Peptic ulcer disease	1
Unspecified peptic ulcer with haemorrhage	Peptic ulcer disease	1
Acute duodenal ulcer without mention of complication	Peptic ulcer disease	1
Chronic duodenal ulcer with haemorrhage and perforation	Peptic ulcer disease	1
Closure of perforated duodenal ulcer	Peptic ulcer disease	1
Chronic peptic ulcer NOS	Peptic ulcer disease	1
Stress ulcer NOS	Peptic ulcer disease	1
Acute peptic ulcer unspecified	Peptic ulcer disease	1
Anti-platelet induced gastric ulcer	Peptic ulcer disease	1
Acute gastric ulcer NOS	Peptic ulcer disease	1
Chronic gastric ulcer	Peptic ulcer disease	1
Acute gastric ulcer with obstruction	Peptic ulcer disease	1
Chronic duodenal ulcer NOS	Peptic ulcer disease	1
ULCER PEPTIC	Peptic ulcer disease	1
Chronic peptic ulcer without mention of complication	Peptic ulcer disease	1
Duodenal ulcer - (DU)	Peptic ulcer disease	1
Chronic gastrojejunal ulcer	Peptic ulcer disease	1
Unspecified gastric ulcer without mention of complication	Peptic ulcer disease	1
Chronic gastric ulcer with perforation	Peptic ulcer disease	1
Duodenal erosion	Peptic ulcer disease	1
REPAIR PERFORATED GASTRIC ULCER	Peptic ulcer disease	1
Unspecified peptic ulcer with obstruction	Peptic ulcer disease	1
Closure of gastric ulcer NEC	Peptic ulcer disease	1
Chronic peptic ulcer with obstruction	Peptic ulcer disease	1
ULCER MARGINAL	Peptic ulcer disease	1
Multiple gastric ulcers	Peptic ulcer disease	1
Unspecified gastrojejunal ulcer NOS	Peptic ulcer disease	1
[V]Personal history of peptic ulcer	Peptic ulcer disease	1
Acute peptic ulcer with haemorrhage	Peptic ulcer disease	1
Resection of gastric ulcer by cautery	Peptic ulcer disease	1
Acute duodenal ulcer with obstruction	Peptic ulcer disease	1
[V] Personal history of gastric ulcer	Peptic ulcer disease	1
ULCER PEPTIC STOMACH	Peptic ulcer disease	1

Read/OXMIS term	Disease category	Charlson score weight
Unspecified duodenal ulcer with haemorrhage and perforation	Peptic ulcer disease	1
Unspecified peptic ulcer NOS	Peptic ulcer disease	1
Stomach ulcer operations	Peptic ulcer disease	1
ULCER DUODENUM PERFORATED	Peptic ulcer disease	1
Perforated chronic gastric ulcer	Peptic ulcer disease	1
Duodenal ulcer NOS	Peptic ulcer disease	1
Acute gastrojejunal ulcer with perforation	Peptic ulcer disease	1
Chronic peptic ulcer with haemorrhage	Peptic ulcer disease	1
Chronic gastrojejunal ulcer without mention of complication	Peptic ulcer disease	1
Chronic peptic ulcer with perforation	Peptic ulcer disease	1
Recurrent duodenal ulcer	Peptic ulcer disease	1
REPAIR PERFORATED PEPTIC ULCER	Peptic ulcer disease	1
Acute peptic ulcer with haemorrhage and perforation	Peptic ulcer disease	1
Unspecified gastric ulcer with haemorrhage and perforation	Peptic ulcer disease	1
Chronic gastrojejunal ulcer with haemorrhage	Peptic ulcer disease	1
Bleeding chronic duodenal ulcer	Peptic ulcer disease	1
ULCER PREPYLORIC	Peptic ulcer disease	1
Other specified operation on gastric ulcer	Peptic ulcer disease	1
Acute gastrojejunal ulcer NOS	Peptic ulcer disease	1
Acute gastrojejunal ulcer unspecified	Peptic ulcer disease	1
Unspecified peptic ulcer with perforation	Peptic ulcer disease	1
Acute gastric ulcer with haemorrhage	Peptic ulcer disease	1
ULCER STOMACH	Peptic ulcer disease	1
DUODENAL ULCER BLEEDING	Peptic ulcer disease	1
Operations on gastric ulcer	Peptic ulcer disease	1
Acute gastrojejunal ulcer	Peptic ulcer disease	1
Juxtarenal aortic aneurysm	Peripheral vascular disease	1
Tube graft abdominal Aortic aneurysm (emergency)	Peripheral vascular disease	1
[D]Gangrene	Peripheral vascular disease	1
Ruptured aortic aneurysm NOS	Peripheral vascular disease	1
H/O: aortic aneurysm	Peripheral vascular disease	1
Other peripheral vascular disease	Peripheral vascular disease	1
[X]Aortic aneurysm of unspecified site, ruptured	Peripheral vascular disease	1
Dissecting aortic aneurysm	Peripheral vascular disease	1
Other specified peripheral vascular disease	Peripheral vascular disease	1
Presenile gangrene	Peripheral vascular disease	1
Ruptured suprarenal aortic aneurysm	Peripheral vascular disease	1

Read/OXMIS term	Disease category	Charlson score weight
Thoracoabdominal aortic aneurysm, ruptured	Peripheral vascular disease	1
Aortic aneurysm	Peripheral vascular disease	1
[D]Gangrene of toe in diabetic	Peripheral vascular disease	1
Gangrene of toe	Peripheral vascular disease	1
INTERMITTENT CLAUDICATION	Peripheral vascular disease	1
Peripheral gangrene	Peripheral vascular disease	1
Thoracoabdominal aortic aneurysm, without mention of rupture	Peripheral vascular disease	1
Gangrene of hand	Peripheral vascular disease	1
GANGRENE TOE	Peripheral vascular disease	1
Emergency repair of aortic aneurysm	Peripheral vascular disease	1
Y graft abdominal Aortic aneurysm	Peripheral vascular disease	1
Other specified peripheral vascular disease NOS	Peripheral vascular disease	1
Diabetes with gangrene	Peripheral vascular disease	1
GANGRENE FOOT	Peripheral vascular disease	1
SCROTAL GANGRENE	Peripheral vascular disease	1
[D]Widespread diabetic foot gangrene	Peripheral vascular disease	1
O/E - gangrene	Peripheral vascular disease	1
Inflammatory abdominal aortic aneurysm	Peripheral vascular disease	1
Gangrene of foot	Peripheral vascular disease	1
Gangrene of finger	Peripheral vascular disease	1
[D]Gangrene NOS	Peripheral vascular disease	1
Intermittent claudication	Peripheral vascular disease	1
Aortic aneurysm without mention of rupture NOS	Peripheral vascular disease	1
DISSECTION AORTA	Peripheral vascular disease	1
Claudication	Peripheral vascular disease	1
Y graft of abdominal Aortic aneurysm (emergency)	Peripheral vascular disease	1
Thoracic aortic aneurysm without mention of rupture	Peripheral vascular disease	1
Peripheral ischaemic vascular disease	Peripheral vascular disease	1

Read/OXMIS term	Disease category	Charlson score weight
ULCER WITH GANGRENE	Peripheral vascular disease	1
Thoracic aortic aneurysm which has ruptured	Peripheral vascular disease	1
Peripheral vascular disease NOS	Peripheral vascular disease	1
Peripheral vascular disease NOS	Peripheral vascular disease	1
Aortic aneurysm NOS	Peripheral vascular disease	1
Abdominal aortic aneurysm which has ruptured	Peripheral vascular disease	1
Aortic aneurysm repair	Peripheral vascular disease	1
AORTIC ANEURYSM	Peripheral vascular disease	1
H/O: Peripheral vascular disease procedure	Peripheral vascular disease	1
Aortic aneurysm	Peripheral vascular disease	1
Ruptured abdominal aortic aneurysm	Peripheral vascular disease	1
[X]Aortic aneurysm of unspecified site, nonruptured	Peripheral vascular disease	1
Gangrene of thumb	Peripheral vascular disease	1
[D]Gangrene, spreading cutaneous	Peripheral vascular disease	1
AAA - Abdominal aortic aneurysm without mention of rupture	Peripheral vascular disease	1
Abdominal aortic aneurysm without mention of rupture	Peripheral vascular disease	1
[X]Other specified peripheral vascular diseases	Peripheral vascular disease	1
Leaking abdominal aortic aneurysm	Peripheral vascular disease	1
Tube graft of Abdominal aortic aneurysm	Peripheral vascular disease	1
PVD (PERIPHERAL VASCULAR DISEASE)	Peripheral vascular disease	1
GANGRENE	Peripheral vascular disease	1
SYMMETRICAL GANGRENE EXTREMITIES	Peripheral vascular disease	1
Ruptured thoracic aortic aneurysm	Peripheral vascular disease	1
RENAL MEDULLARY NECROSIS	Renal disease	2
Phosphate-losing tubular disorders	Renal disease	2
Nephritis unsp????? glomerulonephritis lesion NOS	Renal disease	2
Renal dwarfism	Renal disease	2
MESANGIOCAPILLARY GLOMERULONEPHRITIS	Renal disease	2
Acute renal failure NOS	Renal disease	2
OSTEODYSTROPHY URAEMIC	Renal disease	2
Acute pyelonephritis with medullary necrosis	Renal disease	2

Read/OXMIS term	Disease category	Charlson score weight
Chronic glomerulonephritis NOS	Renal disease	2
Impaired renal function	Renal disease	2
Chronic membranous glomerulonephritis	Renal disease	2
Chronic rapidly progressive glomerulonephritis	Renal disease	2
RENAL DISEASE	Renal disease	2
MEMBRANOPROLIFERATIVE GLOMERULONEPHRITIS	Renal disease	2
Nephritis unsp membranoprolif glomerulonephritis lesion	Renal disease	2
Chronic kidney disease stage 2	Renal disease	2
NEPHRITIS	Renal disease	2
Acute renal cortical necrosis	Renal disease	2
Mesangioproliferative glomerulonephritis NEC	Renal disease	2
GLOMERULONEPHRITIS CHRONIC	Renal disease	2
Nephritis, nephrosis and nephrotic syndrome	Renal disease	2
Nephropathy - chronic	Renal disease	2
Other impaired renal function disorder NOS	Renal disease	2
OSTEODYSTROPHY AZOTAEMIC	Renal disease	2
NEPHROPATHY MEMBRANOUS	Renal disease	2
Nephrogenic diabetes insipidus	Renal disease	2
Renal osteodystrophy	Renal disease	2
Chron neph syn difuse endocap prolifrativ glomerulonephritis	Renal disease	2
Renal failure unspecified	Renal disease	2
Chron nephritic syndrom difuse membranous glomerulonephritis	Renal disease	2
Renal cortical necrosis unspecified	Renal disease	2
Chronic focal glomerulonephritis	Renal disease	2
CHRONIC RENAL FAILURE	Renal disease	2
NEPHRITIS HEREDITARY	Renal disease	2
Nephritis and nephropathy unspecified	Renal disease	2
Chronic renal failure	Renal disease	2
[X]Heredtry nephrpthy NEC difus mesangiocapilry glomneph	Renal disease	2
Acute pyelonephritis without medullary necrosis	Renal disease	2
Chronic glomerulonephritis	Renal disease	2
Renal rickets	Renal disease	2
OSTEODYSTROPHY RENAL	Renal disease	2
NEPHROPATHY HYPOKALAEMIC	Renal disease	2
Chronic kidney disease stage 4	Renal disease	2
Membranoproliferative nephritis unspecified	Renal disease	2
Nephrotic syndrome??????? glomerulonephritis	Renal disease	2
Chron neph syn difus mesangial prolifrtiv glomerulonephritis	Renal disease	2
Chronic kidney disease stage 5	Renal disease	2
[X]Renal failure	Renal disease	2
Impaired renal function disorder NOS	Renal disease	2
End stage renal failure	Renal disease	2

Read/OXMIS term	Disease category	Charlson score weight
RENAL FAILURE	Renal disease	2
Chronic pyelonephritis without medullary necrosis	Renal disease	2
Other acute renal failure	Renal disease	2
H/O: nephritis	Renal disease	2
Nephritis - chronic	Renal disease	2
Chronic kidney disease stage 1	Renal disease	2
Focal membranoproliferative glomerulonephritis	Renal disease	2
CHRONIC NEPHRITIS	Renal disease	2
Nephritis and nephropathy unspecified	Renal disease	2
Acute renal medullary necrosis	Renal disease	2
Chronic membranoproliferative glomerulonephritis	Renal disease	2
Mesangiocapillary glomerulonephritis NEC	Renal disease	2
Renal infantilism	Renal disease	2
Acute nephritis with lesions of necrotising glomerulitis	Renal disease	2
Chronic kidney disease stage 3	Renal disease	2
Other chronic glomerulonephritis NOS	Renal disease	2
Chronic diffuse glomerulonephritis	Renal disease	2
Renal osteodystrophy NOS	Renal disease	2
Nephrotic syn,diffuse mesangiocapillary glomerulonephritis	Renal disease	2
Renal medullary necrosis unspecified	Renal disease	2
Chronic nephritic syn diffuse crescentic glomerulonephritis	Renal disease	2
Hypokalaemic nephropathy	Renal disease	2
[X]Other chronic renal failure	Renal disease	2
Chronic neph syn difus mesangiocapillary glomerulonephritis	Renal disease	2
Chronic glomerulonephritis diseases EC	Renal disease	2
Chronic pyelonephritis with medullary necrosis	Renal disease	2
[X]Other acute renal failure	Renal disease	2
LUPUS ERYTHEMATOSUS SYSTEMIC	Rheumatological disease	1
Felty's syndrome	Rheumatological disease	1
RHEUMATISM	Rheumatological disease	1
RHEUMATISM NONARTICULAR	Rheumatological disease	1
RHEUMATOID ARTHRITIS	Rheumatological disease	1
[X]Rheumatoid arthritis?????? organs or systems	Rheumatological disease	1
Systemic sclerosis induced by drugs and chemicals	Rheumatological disease	1
Caplan's syndrome	Rheumatological disease	1
RHEUMATOID ARTHRITIS SPINE	Rheumatological disease	1
Progressive systemic sclerosis	Rheumatological disease	1

Read/OXMIS term	Disease category	Charlson score weight
Systemic sclerosis	Rheumatological disease	1
Rheumatoid arthritis of hip	Rheumatological disease	1
Systemic lupus erythematosus with organ or sys involv	Rheumatological disease	1
Fibrosing alveolitis associated with rheumatoid arthritis	Rheumatological disease	1
Seropositive errosive rheumatoid arthritis	Rheumatological disease	1
Systemic lupus erythematosus	Rheumatological disease	1
Rheumatoid arthritis of other tarsal joint	Rheumatological disease	1
LUPUS ERYTHEMATOSUS ACUTE	Rheumatological disease	1
Seropositive rheumatoid arthritis, unspecified	Rheumatological disease	1
[X]Seropositive rheumatoid arthritis, unspecified	Rheumatological disease	1
Rheumatoid arthritis of DIP joint of finger	Rheumatological disease	1
Rheumatoid arthritis of sternoclavicular joint	Rheumatological disease	1
Rheumatism unspecified	Rheumatological disease	1
Muscular rheumatism	Rheumatological disease	1
SERO POSITIVE RHEUMATOID ARTHRITIS	Rheumatological disease	1
Giant cell arteritis with polymyalgia rheumatica	Rheumatological disease	1
Polymyalgia rheumatica	Rheumatological disease	1
SCLERODERMA DIFFUSE	Rheumatological disease	1
Rheumatoid arthritis of PIP joint of finger	Rheumatological disease	1
Libman-Sacks disease	Rheumatological disease	1
SYSTEMIC LUPUS ERYTHEMATOSUS WITH RENAL	Rheumatological disease	1
Systemic lupus erythematosus with pericarditis	Rheumatological disease	1
Rheumatoid arthritis of IP joint of toe	Rheumatological disease	1
Rheumatoid arthritis of MCP joint	Rheumatological disease	1
Other specified nonarticular rheumatism	Rheumatological disease	1
Lung disease with systemic sclerosis	Rheumatological disease	1
Rheumatoid arthritis and other inflammatory polyarthropathy	Rheumatological disease	1
Seronegative rheumatoid arthritis	Rheumatological disease	1

Read/OXMIS term	Disease category	Charlson score weight
RHEUMATISM MUSCULAR ARM	Rheumatological disease	1
Rheumatoid carditis	Rheumatological disease	1
SCLERODERMA ACROSCLEROTIC	Rheumatological disease	1
Lung disease with systemic lupus erythematosus	Rheumatological disease	1
Lupus nephritis	Rheumatological disease	1
Acrosclerosis	Rheumatological disease	1
POLYMYOSITIS	Rheumatological disease	1
RHEUMATOID ARTHRITIS INCREASED ACTIVITY	Rheumatological disease	1
Disseminated lupus erythematosus	Rheumatological disease	1
Flare of rheumatoid arthritis	Rheumatological disease	1
Rheumatoid arthritis	Rheumatological disease	1
Sero negative polyarthritis	Rheumatological disease	1
[X]Other forms of systemic sclerosis	Rheumatological disease	1
SERO NEGATIVE RHEUMATOID ARTHRITIS	Rheumatological disease	1
Rheumatism or fibrositis NOS	Rheumatological disease	1
SYNDROME FELTY'S	Rheumatological disease	1
RHEUMATISM HANDS	Rheumatological disease	1
Rheumatoid arthritis of lesser MTP joint	Rheumatological disease	1
Polymyositis ossificans	Rheumatological disease	1
SCLERODERMA	Rheumatological disease	1
[X]Other specified rheumatoid arthritis	Rheumatological disease	1
MONOARTICULAR RHEUMATISM	Rheumatological disease	1
Myopathy due to scleroderma	Rheumatological disease	1
Myopathy due to rheumatoid arthritis	Rheumatological disease	1
Rheumatoid lung disease	Rheumatological disease	1
Rheumatoid arthritis of subtalar joint	Rheumatological disease	1
LIBMAN- SACKS DISEASE	Rheumatological disease	1
Rheumatoid arthritis of shoulder	Rheumatological disease	1

Read/OXMIS term	Disease category	Charlson score weight
Rheumatoid arthritis of talonavicular joint	Rheumatological disease	1
Rheumatoid arthritis of knee	Rheumatological disease	1
RHEUMATISM HANDS ACUTE	Rheumatological disease	1
Rheumatoid arthritis of ankle	Rheumatological disease	1
Endemic polyarthritis	Rheumatological disease	1
[X]Other forms of systemic lupus erythematosus	Rheumatological disease	1
[X]Other seropositive rheumatoid arthritis	Rheumatological disease	1
RHEUMATISM NONARTICULAR SHOULDER	Rheumatological disease	1
Polymyositis	Rheumatological disease	1
Lung disease with polymyositis	Rheumatological disease	1
Rheumatoid arthritis of tibio-fibular joint	Rheumatological disease	1
Rheumatoid arthritis of distal radio-ulnar joint	Rheumatological disease	1
Rheumatism and fibrositis unspecified	Rheumatological disease	1
Rheumatoid arthritis of sacro-iliac joint	Rheumatological disease	1
Myopathy due to disseminated lupus erythematosus	Rheumatological disease	1
LUPUS ERYTHEMATOSUS DISSEMINATED	Rheumatological disease	1
SCLERODERMA GENERALIZED	Rheumatological disease	1
Rheumatoid arthritis of 1st MTP joint	Rheumatological disease	1
Scleroderma	Rheumatological disease	1
Nonarticular rheumatism NOS	Rheumatological disease	1
POLYMYALGIA RHEUMATICA	Rheumatological disease	1
Rheumatoid arthritis of elbow	Rheumatological disease	1
Drug-induced systemic lupus erythematosus	Rheumatological disease	1
Nephrotic syndrome in systemic lupus erythematosus	Rheumatological disease	1
Rheumatoid lung	Rheumatological disease	1
Systemic lupus erythematosus NOS	Rheumatological disease	1
Rheumatoid arthritis of wrist	Rheumatological disease	1
Hand rheumatism	Rheumatological disease	1

Read/OXMIS term	Disease category	Charlson score weight
RHEUMATISM MUSCULAR	Rheumatological disease	1
Rheumatoid arthritis of acromioclavicular joint	Rheumatological disease	1
Rheumatoid lung	Rheumatological disease	1
ACROSCLEROSIS	Rheumatological disease	1
ACUTE SYSTEMIC LUPUS ERYTHEMATOSUS	Rheumatological disease	1
Polyneuropathy in rheumatoid arthritis	Rheumatological disease	1
SYSTEMIC LUPUS ERYTHEMATOSUS	Rheumatological disease	1
RHEUMATIC ARTHRITIS	Rheumatological disease	1

Table S8. ICD-10 codes for identifying comorbidities to calculate the Charlson comorbidity index

ICD-10	Description	weight
I25.2	Myocardial infarction	1
I09.9	Congestive heart failure	1
I11.0	Congestive heart failure	1
I13.0	Congestive heart failure	1
I13.2	Congestive heart failure	1
I25.5	Congestive heart failure	1
I42.0	Congestive heart failure	1
I42.5	Congestive heart failure	1
I42.6	Congestive heart failure	1
I42.7	Congestive heart failure	1
I42.8	Congestive heart failure	1
I42.9	Congestive heart failure	1
P29.0	Congestive heart failure	1
I73.1	Peripheral vascular disease	1
I73.8	Peripheral vascular disease	1
I73.9	Peripheral vascular disease	1
I77.1	Peripheral vascular disease	1
I79.0	Peripheral vascular disease	1
I79.2	Peripheral vascular disease	1
K55.1	Peripheral vascular disease	1
K55.8	Peripheral vascular disease	1
K55.9	Peripheral vascular disease	1
Z95.8	Peripheral vascular disease	1
Z95.9	Peripheral vascular disease	1
H34.0	Cerebrovascular disease	1
F05.1	Dementia	1
G31.1	Dementia	1
I27.8	Chronic pulmonary disease	1
I27.9	Chronic pulmonary disease	1
J68.4	Chronic pulmonary disease	1
J70.1	Chronic pulmonary disease	1
J70.3	Chronic pulmonary disease	1
M31.5	Rheumatic disease	1
M35.1	Rheumatic disease	1
M35.3	Rheumatic disease	1
M36.0	Rheumatic disease	1
K70.0	Mild liver disease	1
K70.1	Mild liver disease	1
K70.2	Mild liver disease	1
K70.3	Mild liver disease	1
K70.9	Mild liver disease	1
K71.3	Mild liver disease	1
K71.4	Mild liver disease	1
K71.5	Mild liver disease	1

K71.7	Mild liver disease	1
K76.0	Mild liver disease	1
K76.2	Mild liver disease	1
K76.3	Mild liver disease	1
K76.4	Mild liver disease	1
K76.8	Mild liver disease	1
K76.9	Mild liver disease	1
Z94.4	Mild liver disease	1
E10.0	Diabetes without chronic complication	1
E10.1	Diabetes without chronic complication	1
E10.6	Diabetes without chronic complication	1
E10.8	Diabetes without chronic complication	1
E10.9	Diabetes without chronic complication	1
E11.0	Diabetes without chronic complication	1
E11.1	Diabetes without chronic complication	1
E11.6	Diabetes without chronic complication	1
E11.8	Diabetes without chronic complication	1
E11.9	Diabetes without chronic complication	1
E12.0	Diabetes without chronic complication	1
E12.1	Diabetes without chronic complication	1
E12.6	Diabetes without chronic complication	1
E12.8	Diabetes without chronic complication	1
E12.9	Diabetes without chronic complication	1
E13.0	Diabetes without chronic complication	1
E13.1	Diabetes without chronic complication	1
E13.6	Diabetes without chronic complication	1
E13.8	Diabetes without chronic complication	1
E13.9	Diabetes without chronic complication	1
E14.0	Diabetes without chronic complication	1
E14.1	Diabetes without chronic complication	1
E14.6	Diabetes without chronic complication	1
E14.8	Diabetes without chronic complication	1
E14.9	Diabetes without chronic complication	1
E10.2	Diabetes with chronic complication	2
E10.3	Diabetes with chronic complication	2
E10.4	Diabetes with chronic complication	2
E10.5	Diabetes with chronic complication	2
E10.7	Diabetes with chronic complication	2
E11.2	Diabetes with chronic complication	2
E11.3	Diabetes with chronic complication	2
E11.4	Diabetes with chronic complication	2
E11.5	Diabetes with chronic complication	2
E11.7	Diabetes with chronic complication	2
E12.2	Diabetes with chronic complication	2
E12.3	Diabetes with chronic complication	2
E12.4	Diabetes with chronic complication	2
E12.5	Diabetes with chronic complication	2

E12.7	Diabetes with chronic complication	2
E13.2	Diabetes with chronic complication	2
E13.3	Diabetes with chronic complication	2
E13.4	Diabetes with chronic complication	2
E13.5	Diabetes with chronic complication	2
E13.7	Diabetes with chronic complication	2
E14.2	Diabetes with chronic complication	2
E14.3	Diabetes with chronic complication	2
E14.4	Diabetes with chronic complication	2
E14.5	Diabetes with chronic complication	2
E14.7	Diabetes with chronic complication	2
G04.1	Hemiplegia or paraplegia	2
G11.4	Hemiplegia or paraplegia	2
G80.1	Hemiplegia or paraplegia	2
G80.2	Hemiplegia or paraplegia	2
G83.0	Hemiplegia or paraplegia	2
G83.1	Hemiplegia or paraplegia	2
G83.2	Hemiplegia or paraplegia	2
G83.3	Hemiplegia or paraplegia	2
G83.9	Hemiplegia or paraplegia	2
I12.0	Renal disease	2
I13.1	Renal disease	2
N03.2	Renal disease	2
N03.3	Renal disease	2
N03.4	Renal disease	2
N03.5	Renal disease	2
N03.6	Renal disease	2
N03.7	Renal disease	2
N05.2	Renal disease	2
N05.3	Renal disease	2
N05.4	Renal disease	2
N05.5	Renal disease	2
N05.6	Renal disease	2
N05.7	Renal disease	2
N25.0	Renal disease	2
Z49.0	Renal disease	2
Z49.1	Renal disease	2
Z49.2	Renal disease	2
Z94.0	Renal disease	2
Z99.2	Renal disease	2
I85.0	Moderate or severe liver disease	2
I85.9	Moderate or severe liver disease	2
I86.4	Moderate or severe liver disease	2
I98.2	Moderate or severe liver disease	2
K70.4	Moderate or severe liver disease	2
K71.1	Moderate or severe liver disease	2
K72.1	Moderate or severe liver disease	2

K72.9	Moderate or severe liver disease	2
K76.5	Moderate or severe liver disease	2
K76.6	Moderate or severe liver disease	2
K76.7	Moderate or severe liver disease	2

Table S9. ICD-10 codes for frailty score

ICD-10 code	Description	Score
F00	Dementia in Alzheimer's disease	7.1
G81	Hemiplegia	4.4
G30	Alzheimer's disease	4
I69	Sequelae of cerebrovascular disease (secondary codes)	3.7
R29	Other symptoms and signs involving the nervous and musculoskeletal systems (R29??6 Tendency to fall)	3.6
N39	Other disorders of urinary system (includes urinary tract infection and urinary incontinence)	3.2
F05	Delirium, not induced by alcohol and other psychoactive substances	3.2
W19	Unspecified fall	3.2
S00	Superficial injury of head	3.2
R31	Unspecified haematuria	3
B96	Other bacterial agents as the cause of diseases classified to other chapters (secondary code)	2.9
R41	Other symptoms and signs involving cognitive functions and awareness	2.7
R26	Abnormalities of gait and mobility	2.6
I67	Other cerebrovascular diseases	2.6
R56	Convulsions, not elsewhere classified	2.6
R40	Somnolence, stupor and coma	2.5
T83	Complications of genitourinary prosthetic devices, implants and grafts	2.4
S06	Intracranial injury	2.4
S42	Fracture of shoulder and upper arm	2.3
E87	Other disorders of fluid, electrolyte and acid- base balance	2.3
M25	Other joint disorders, not elsewhere classified	2.3
E86	Volume depletion	2.3
R54	Senility	2.2
Z50	Care involving use of rehabilitation procedures	2.1
F03	Unspecified dementia	2.1
W18	Other fall on same level	2.1
Z75	Problems related to medical facilities and other health care	2
F01	Vascular dementia	2
S80	Superficial injury of lower leg	2
L03	Cellulitis	2
H54	Blindness and low vision	1.9
E53	Deficiency of other B group vitamins	1.9
Z60	Problems related to social environment	1.8
G20	Parkinson's disease	1.8
R55	Syncope and collapse	1.8
S22	Fracture of rib(s), sternum and thoracic spine	1.8
K59	Other functional intestinal disorders	1.8
N17	Acute renal failure	1.8
L89	Decubitus ulcer	1.7
Z22	Carrier of infectious disease	1.7
B95	Streptococcus and staphylococcus as the cause of diseases classified to other chapters	1.7

L97	Ulcer of lower limb, not elsewhere classified	1.6
R44	Other symptoms and signs involving general sensations and perceptions	1.6
K26	Duodenal ulcer	1.6
I95	Hypotension	1.6
N19	Unspecified renal failure	1.6
A41	Other septicaemia	1.6
Z87	Personal history of other diseases and conditions	1.5
J96	Respiratory failure, not elsewhere classified	1.5
X59	Exposure to unspecified factor	1.5
M19	Other arthrosis	1.5
G40	Epilepsy	1.5
M81	Osteoporosis without pathological fracture	1.4
S72	Fracture of femur	1.4
S32	Fracture of lumbar spine and pelvis	1.4
E16	Other disorders of pancreatic internal secretion	1.4
R94	Abnormal results of function studies	1.4
N18	Chronic renal failure	1.4
R33	Retention of urine	1.3
R69	Unknown and unspecified causes of morbidity	1.3
N28	Other disorders of kidney and ureter, not elsewhere classified	1.3
R32	Unspecified urinary incontinence	1.2
G31	Other degenerative diseases of nervous system, not elsewhere classified	1.2
Y95	Nosocomial condition	1.2
S09	Other and unspecified injuries of head	1.2
R45	Symptoms and signs involving emotional state	1.2
G45	Transient cerebral ischaemic attacks and related syndromes	1.2
Z74	Problems related to care-provider dependency	1.1
M79	Other soft tissue disorders, not elsewhere classified	1.1
W06	Fall involving bed	1.1
S01	Open wound of head	1.1
A04	Other bacterial intestinal infections	1.1
A09	Diarrhoea and gastroenteritis of presumed infectious origin	1.1
J18	Pneumonia, organism unspecified	1.1
J69	Pneumonitis due to solids and liquids	1
R47	Speech disturbances, not elsewhere classified	1
E55	Vitamin D deficiency	1
Z93	Artificial opening status	1
R02	Gangrene, not elsewhere classified	1
R63	Symptoms and signs concerning food and fluid intake	0.9
H91	Other hearing loss	0.9
W10	Fall on and from stairs and steps	0.9
W01	Fall on same level from slipping, tripping and stumbling	0.9
E05	Thyrotoxicosis [hyperthyroidism]	0.9
M41	Scoliosis	0.9
R13	Dysphagia	0.8
Z99	Dependence on enabling machines and devices	0.8

U80	Agent resistant to penicillin and related antibiotics	0.8
M80	Osteoporosis with pathological fracture	0.8
K92	Other diseases of digestive system	0.8
I63	Cerebral Infarction	0.8
N20	Calculus of kidney and ureter	0.7
F10	Mental and behavioural disorders due to use of alcohol	0.7
Y84	Other medical procedures as the cause of abnormal reaction of the patient	0.7
R00	Abnormalities of heart beat	0.7
J22	Unspecified acute lower respiratory infection	0.7
Z73	Problems related to life-management difficulty	0.6
R79	Other abnormal findings of blood chemistry	0.6
Z91	Personal history of risk-factors, not elsewhere classified	0.5
S51	Open wound of forearm	0.5
F32	Depressive episode	0.5
M48	Spinal stenosis (secondary code only)	0.5
E83	Disorders of mineral metabolism	0.4
M15	Polyarthrosis	0.4
D64	Other anaemias	0.4
L08	Other local infections of skin and subcutaneous tissue	0.4
R11	Nausea and vomiting	0.3
K52	Other noninfective gastroenteritis and colitis	0.3
R50	Fever of unknown origin	0.1

Table S10. Mean value and standardised mean difference of selected covariates in propensity score before and after balance

Variables	Mean in treat	Mean in untreated	SMD
Age 70-75	0.1	0.1	-0.001
Age 75-80	0.09	0.09	0.000
Age 80-85	0.06	0.06	-0.001
Age 85-90	0.04	0.04	-0.009
Age 90+	0.04	0.04	-0.009
CCI low	0.87	0.87	0.000
CCI intermediate	0.1	0.1	0.002
CCI high	0.03	0.03	-0.003
Non frail	0.92	0.91	0.015
Pre frail	0.06	0.07	-0.008
Frail	0.02	0.02	-0.014
IMD (1-2)	0.23	0.21	0.049
IMD (3-4)	0.19	0.18	0.013
IMD (5-6)	0.16	0.16	-0.006
IMD (7-8)	0.07	0.08	-0.018
IMD (9-10)	0.09	0.11	-0.041

(Note) All variables in propensity score matching were binary variables. SMD: Standardised mean difference; CCI: Charlson Comorbidity Index; IMD: index of multiple deprivation

Table S11. Tumour characteristics of the study cohort

Factors		Younger age		Older age	
		**Surgery (n=33,341)**	**PET (n=2,354)**	**Surgery (n=16,096)**	**PET (n=7,013)**
**Tumour grade**	G1	6788 (20.36%)	137 (5.82%)	2380 (14.79%)	588 (8.38%)
	G2	15908 (47.71%)	346 (14.70%)	7895 (49.05%)	1918 (27.35%)
	G3	5910 (17.73%)	143 (6.07%)	3224 (20.03%)	476 (6.79%)
	GX	1136 (3.41%)	305 (12.96%)	1033 (6.42%)	1921 (27.39%)
	Missing	3599 (10.79%)	1423 (60.45%)	1564 (9.72%)	2110 (30.09%)
**NPI**	Median (IQR)	3.36 (3.14-4.38)	3.31 (3.1-4.22)	3.6 (3.24-4.5)	3.3 (2.4-3.6)
	I	145 (0.43%)	-	73 (0.45%)	5 (0.07%)
	II	1892 (5.67%)	7 (0.30%)	434 (2.70%)	0 (0.00%)
	III	3575 (10.72%)	10 (0.42%)	1039 (6.46%)	7 (0.10%)
	IV	4324 (12.97%)	14 (0.59%)	2057 (12.78%)	9 (0.13%)
	V	1009 (3.03%)	1 (0.04%)	543 (3.37%)	1 (0.01%)
	Missing	22396 (67.17%)	2322 (98.64%)	11950 (74.24%)	6991 (99.69%)
**Her-2 status**	Positive	1512 (4.53%)	29 (1.23%)	553 (3.44%)	132 (1.88%)
	Negative	9837 (29.50%)	172 (7.31%)	3970 (24.66%)	945 (13.47%)
	Unknown	21992 (65.96%)	2153 (91.46%)	11573 (71.90%)	5936 (84.64%)

(Note) PET: primary endocrine therapy; IQR: Interquartile range; NPI: Nottingham prognosis.

Supplementary Section S2. Survival time of study cohort between surgery and PET by levels of frailty

Group	Survival time (Median, IQR)		
	Surgery	PET	P value
**HFRS**			
Non-frail	6.8 (4.0-10.6)	3.2 (1.4-6.0)	<0.0001
Pre frail	4.1 (2.5-6.7)	2.3 (0.9-3.9)	<0.0001
Frail	2.8 (1.6-4.8)	1.7 (0.7-3.0)	<0.0001
**CCI**			
Low CCI	7.0 (4.0-10.9)	3.0 (1.2-5.6)	<0.0001
Intermediate CCI	5.1 (3.1-7.7)	2.5 (1.0-4.4)	<0.0001
High CCI	3.8 (2.2-6.1)	2.3 (1.0-3.8)	<0.0001

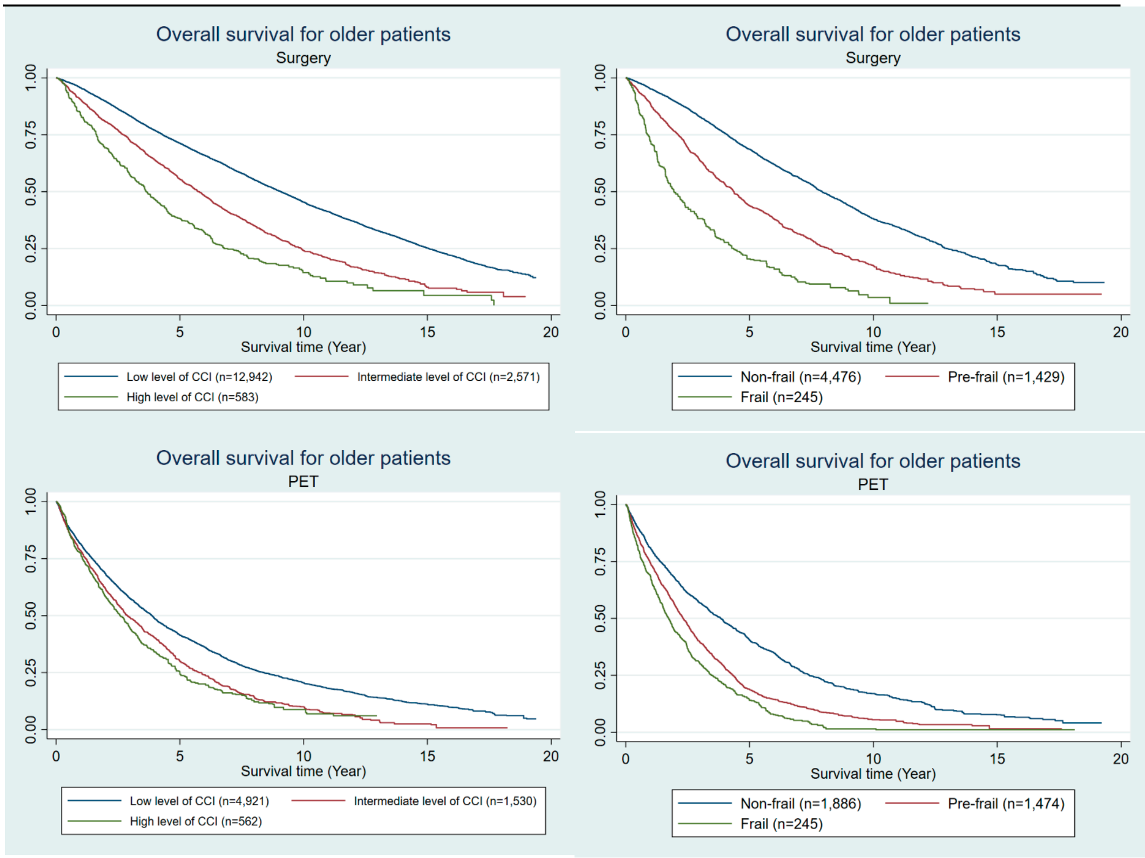

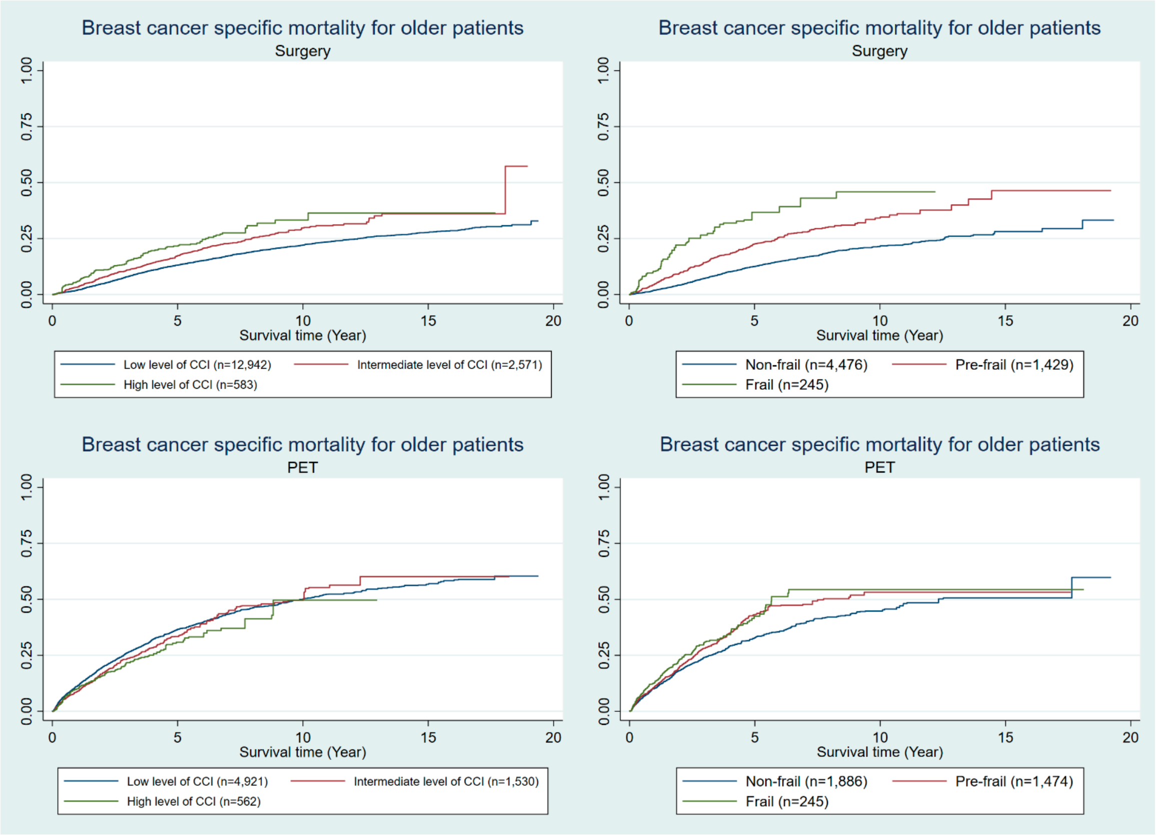

Breast cancer specific mortality for older patients
Surgery
Low level of CCI (n=12,942)
Intermediate level of CCI (n=2,571)
High level of CCI (n=583)
Breast cancer specific mortality for older patients
Surgery
Non-frail (n=4,476)
Pre-frail (n=1,429)
Frail (n=245)
Breast cancer specific mortality for older patients
PET
Low level of CCI (n=4,921)
Intermediate level of CCI (n=1,530)
High level of CCI (n=562)
Breast cancer specific mortality for older patients
PET
Non-frail (n=1,886)
Pre-frail (n=1,474)
Frail (n=245)
Survival time (Year)
0.00
0.25
0.50
0.75
1.00
0
5
10
15
20

Table S12. Competing risk regression of all selected covariates for cohort study

Factors	Model 1		Model 2	
	SHR (95% CI)	P value	SHR (95% CI)	P value
Age	1.1 (1.1, 1.1)	<0.001	1.1 (1.0, 1.1)	<0.001
Age (time-varying)	1.0 (1.0. 1.0)	0.108	1.0 (1.0. 1.0)	0.335
Treatment (reference surgery)				
PET	1.9 (1.8, 2.0)	<0.001	1.2 (1.1, 1.3)	<0.001
HFRS (reference: non-frail)				
Pre-frail	1.4 (1.3, 1.5)	<0.001	1.3 (1.2, 1.4)	<0.001
Frail	2.0 (1.9, 2.2)	<0.001	1.4 (1.2, 1.6)	<0.001
CCI (reference: low level)				
Intermediate	1.3 (1.3, 1.4)	<0.001	1.3 (1.2, 1.4)	<0.001
High	1.8 (1.7, 1.9)	<0.001	1.6 (1.4, 1.8)	<0.001
IMD (reference: IMD decile 1-2)				
3-4	1.0 (1.0, 1.1)	<0.001	1.1 (1.0, 1.2)	0.095
5-6	1.0 (1.0, 1.1)	<0.001	1.1 (1.0, 1.2)	0.125
7-8	1.1 (1.0, 1.2)	<0.001	1.1 (1.0, 1.2)	0.071
9-10	1.2 (1.1, 1.3)	<0.001	1.1 (1.0, 1.2)	0.001
No observations	1.2 (1.1, 1.3)	<0.001	1.1 (1.0, 1.2)	0.032

(Note) HR: hazard ratio; SHR: subdistribution hazard ratio; CI: confidence interval; PET: primary endocrine therapy; HFRS: hospital frailty risk score; CCI: Charlson comorbidity index; IMD: Index of Multiple Deprivation; *Model 1 compared the competing risk between all-cause death and non-cancer cause death; Model 2 compared the competing risk between other cause death and breast cancer-specific death.

Figure S2. Cumulative incidence function of competing risk between PET and surgery in three level of hospital frailty risk scores

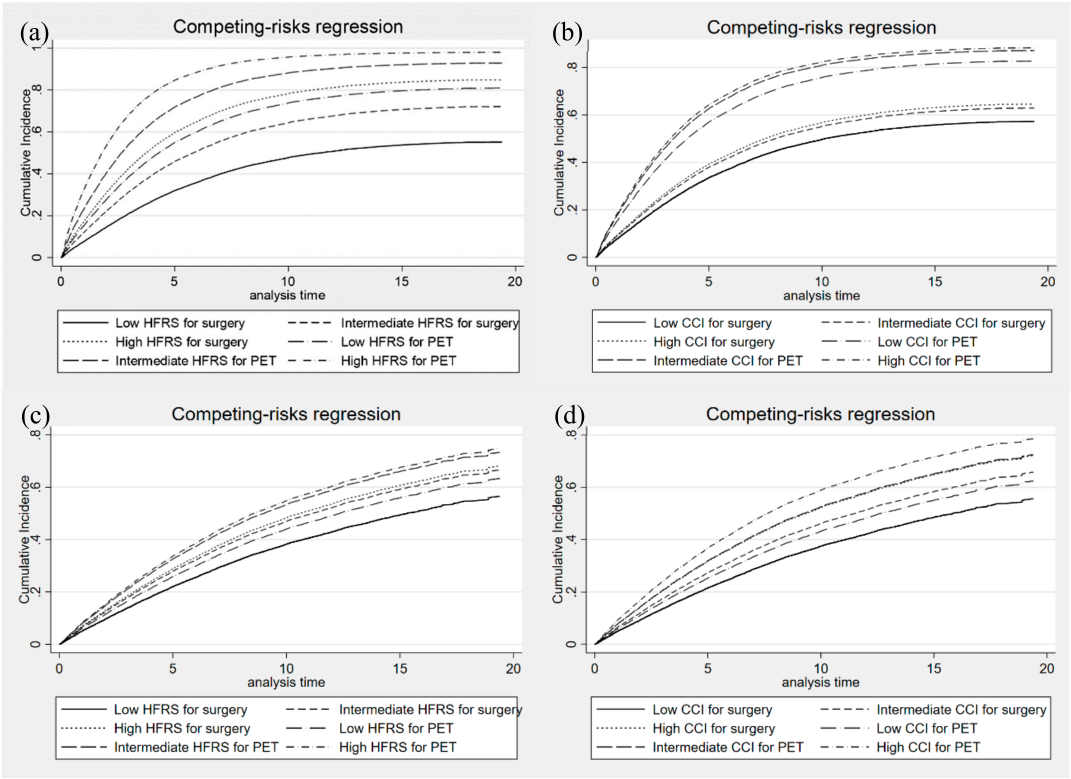

(Note) HFRS: hospital frailty risk score; CCI: Charlson comorbidity index.
*Model 1 compared the competing risk between all-cause death and non-cancer cause death;
Model 2 compared the competing risk between other causes of death and breast cancer-specific death.
(a) Cumulative incidence function between surgery and PET by three levels of HFRS in Model 1;
(b) Cumulative incidence function between surgery and PET by three levels of CCI in Model 1;
(c) Cumulative incidence function between surgery and PET by three levels of HFRS in Model 2;
(d) Cumulative incidence function between surgery and PET by three levels of CCI in Model 2

Figure S3. Cumulative incidence function of competing risk in high level of frailty between surgery and PET

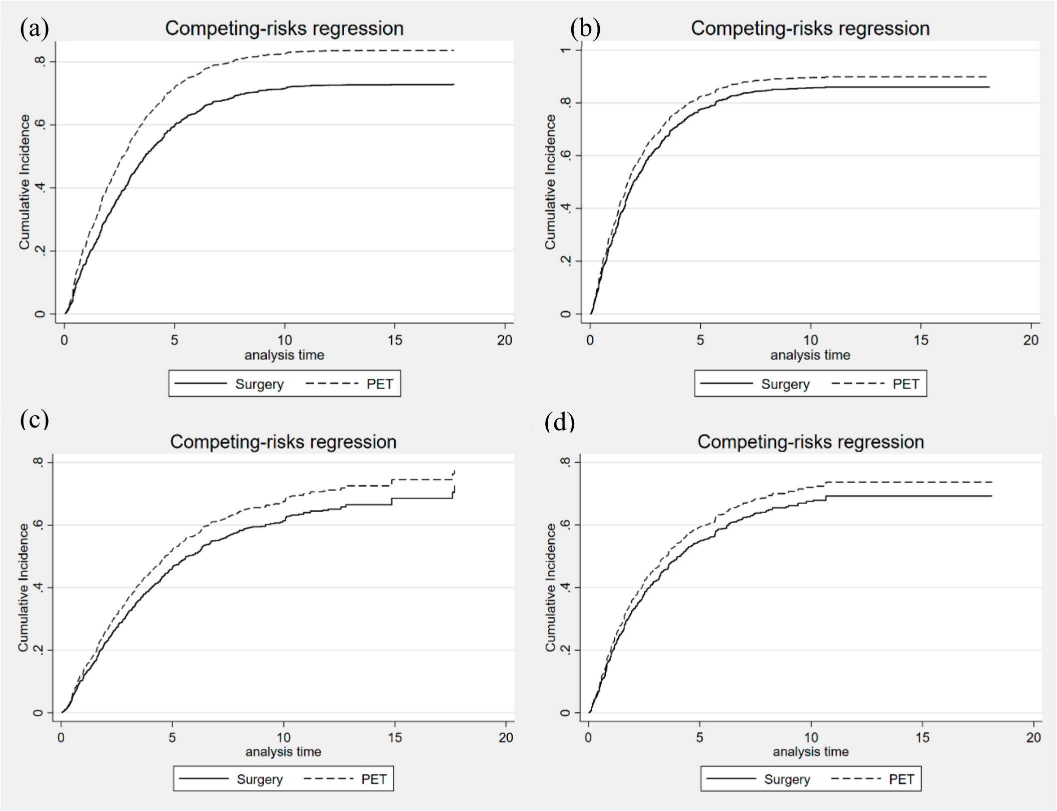

(Note) *Model 1 compared the competing risk between all-cause death and non-cancer cause death; Model 2 compared the competing risk between other cause death and breast cancer specific death.

(a) Cumulative incidence function with high level of CCI by surgery and PET in Model 1; (b) Cumulative incidence function with high level of HFRS by surgery and PET in Model 1 (c) Cumulative incidence function with high level of CCI by surgery and PET in Model 2; (d) Cumulative incidence function with high level of HFRS by surgery and PET in Model 2.
